# A tractable covalent linker strategy for the production of immunogenic antigen-TLR7/8L bioconjugates†

**DOI:** 10.1039/d1cc00795e

**Published:** 2021-05-11

**Authors:** C. J. Massena, S. K. Lathrop, C. J. Davison, R. Schoener, H. G. Bazin, J. T. Evans, D. J. Burkhart

**Affiliations:** Dept. of Biomedical & Pharmaceutical Sciences, University of Montana, 32 Campus Dr, Missoula, MT 59812, USA.

## Abstract

Despite the ease of production and improved safety profiles of recombinant vaccines, the inherently low immunogenicity of unadjuvanted proteins remains an impediment to their widespread adoption. The covalent tethering of TLR agonists to antigenic proteins offers a unique approach to co-deliver both constituents to the same cell–enhancing vaccine efficacy while minimizing reactogenicity. However, the paucity of simple and effective linker chemistries continues to hamper progress. Here, we present a modular, PEG-based linker system compatible with even extremely lipophilic and challenging TLR7/8 agonists. To advance the field and address previous obstacles, we offer the most straightforward and antigen-preserving linker system to date. These antigen-adjuvant conjugates enhance antigen-specific immune responses in mice, demonstrating the power of our approach within the context of modern vaccinology.

In the ongoing effort to improve the efficacy and safety of modern vaccines, direct covalent attachment of Toll-like receptor (TLR) agonists to antigenic proteins has emerged as an effective strategy to enhance immunity while minimizing adjuvant toxicity.^1–3^ TLR7/8 ligands (TLR7/8Ls) in particular have been utilized in this approach due to the broad expression of their receptors in dendritic cells (DCs) and other antigen presenting cells (APCs).^4,5^ In their pioneering work, Wille-Reece *et al*. covalently bound 3M-012 to HIV-1 Gag, ensuring synchronous uptake and processing of both the antigen and adjuvant. This strategy effectively (1) prevented the systemic toxicity associated with the rapid distribution of free and excess TLR agonists, (2) promoted influx and activation of DCs to/in draining lymph nodes (DLNs), and (3) elicited the upregulation of proinflammatory cytokines and costimulatory molecules needed to generate robust, antigen-specific Th1 and CD8^+^ T cell responses.^6–8^ As a general approach, the conjugation of TLR7/8Ls with antigenic proteins has since been extended to viral,^9^ bacterial,^10^ parasitic,^11^ and allergic^12^ disease models, as well as fundamental studies^13–15^ exploring the benefits of protein-adjuvant conjugation. Despite the advantages of this strategy, the practical challenges stemming from the covalent attachment of lipophilic purine- and imidazoquinoline-based agonists to proteins have impeded the advancement of the field.

Previous work involving TLR7/8 bioconjugates largely focused on immunological responses and protection with relatively little attention paid to linker chemistry. The linker methodologies used in these studies suffered from multiple drawbacks, limiting their widespread use, including: photochemical reactions that required high and potentially denaturing pH (9–9.5); liberal use of lipophilic alkyl linkers, contributing to antigen precipitation; two-step conjugations that left behind unused cross-linkers;^9^ and onerous processes that are not amenable to commercial-scale vaccine development (see Table S1, ESI†) for a review of previously reported bioconjugation strategies). In contrast to previous work, we have developed a linker system that is simple, scalable, antigen preserving (with respect to yield and epitopicity), and modular. Given the poor water solubility of many potent TLR7/8Ls as well as the immense biodiversity of recombinant antigens, the need for versatile and effective covalent linker chemistries remains pressing.

Herein, we introduce a modular approach to create protein-TLR7/8L bioconjugates, which elicit robust antigen-specific immune responses *in vivo*. This new system utilizes novel, water-soluble cross-linkers that incorporate polyethylene–glycol (PEG) spacers of any chain-length needed to offset the lipophilicity typical of many TLR7/8Ls.^16^ Additionally, through the isolation and use of lysine-reactive *N*-hydroxysuccinimide (NHS) esters, a single conjugation step affords an adjuvant-to-protein molar ratio (copy #) that is tunable and easy to characterize. Previously developed by our group, two synthetic oxoadenines (UM-3006 and UM-3007)^17,18^ and one synthetic imidazoquinoline (UM-3013, see ESI† Section 2 for characterization data) were selected because they possess amine nucleophilic handles and exhibit moderate (UM-3007), sparing (UM-3013), and negligible (UM-3006) water solubilities upon amide functionalization (see Fig. 1A for structures and ESI† Section 3, Table S2 for comparative solubilities). Typically, ligand solubility correlates with antigen yield during conjugation; thus, these compounds served as a suitable test group for our new linker platform. Our efforts culminated with the high-yield production of conjugate **C4** (Fig. 2A), functionalized with our most potent^18^ yet least water-soluble TLR7/8L, UM-3006 (the relative *in vitro* potencies of these TLR7/8Ls are shown in Fig. S11, ESI†).

Our first attempts to produce an antigen-UM-3006 bioconjugate involved the use of Traut’s reagent to synthesize compound **1** (Fig. 1B). Upon nucleophilic attack and transient ring opening, Traut’s reagent provided a terminal thiol and compensatory cationic amidinium—restoring the solubility-imparting formal charge lost upon amide functionalization of the piperidyl amine. We created conjugate **C1** consisting of a model antigen, cross-reacting material 197 (CRM-197), coupled with compound **1** in two bioconjugation steps (Fig. 2A). First, an aliphatic heterobifunctional cross-linker, *N*-γ-maleimidobutyryl-oxysuccinimide ester (GMBS)—commonly used to append thiol-reactive maleimides to lysines—was added to CRM-197 in 10 mM phosphate buffer (PB) pH 7.2 to create CRM-197-**R2** with an average initial GMBS copy # of 8.7. Post-ultrafiltration, CRM-197-**R2** was reacted with *in situ* formed compound **1**, achieving a UM-3006 copy # of 4.4 (leaving 4.3 unreacted GMBS groups; see Table 1 for a summary of bioconjugation data). This process resulted in a highly immunogenic self-adjuvanting bioconjugate (*vide infra*). However, poor bioconjugation yield (26% antigen recovery, Table 1) and the introduction of 4.3 residual GMBS cross-linkers (with the potential for epitope masking) motivated the development of a more efficient bioconjugation process.

Initial efforts to improve conjugation efficiency focused on replacing GMBS with a PEG-based heterobifunctional cross-linker. If successful, the modular use of hydrophilic PEG linkers could reduce antigen precipitation during conjugation and improve yields. Initially, we employed our most synthetically tractable agonist, UM-3007, which exhibits moderate water solubility when amide functionalized. Analogous to **1**, compound **2** was synthesized through the addition of Traut’s reagent (Fig. 1B). Compound **2** was stabilized and isolated via reaction acidification,^19,20^ prep-HPLC purification, and lyophilization (see ESI† Section 2). To the best of our knowledge, this is the first reported preparatory-scale procedure for isolating a Traut’s derivative of a primary amine in high yield (85%). Unfortunately, this protocol could not be applied to UM-3006, which possesses a secondary amine. Next, we assembled conjugate **C2**—consisting of the more soluble model antigen bovine serum albumin (BSA) decorated with succinimidyl-[(*N*-maleimidopropionamido)-diethyleneglycol] ester (SM(PEG)_2_) and compound **2** (Fig. 2A)—in two bioconjugation steps. First, BSA was functionalized with SM(PEG)_2_ in 10 mM PB pH 7.2 then ultrafiltered to afford a maleimide-functionalized intermediate (BSA-**R4**; average initial SM(PEG)_2_ copy # of 8.3). Subsequently, compound **2** was added in molar excess to BSA-**R4**, resulting in conjugate **C2** with a UM-3007 copy # of 4.4 (leaving 3.9 unreacted SM(PEG)_2_ groups).

We hypothesized that this self-adjuvanting bioconjugate would induce a superior immune response against the model antigen BSA compared to unconjugated controls. Briefly, mice were vaccinated twice, 14 days apart, with 1 or 10 μg of BSA either alone, conjugated with UM-3007 (**C2**), or mixed with free UM-3007 at the corresponding molar ratio. Fourteen days after the second vaccination, BSA-specific total IgG serum antibody titers were measured by ELISA (for experimental details, see ESI† Section 5). Conjugate **C2** induced a significantly higher anti-BSA serum antibody response with an average total IgG titer more than 14 times greater than that of mice vaccinated with unconjugated antigen and adjuvant (Fig. S14, ESI†). Together, the successful production of conjugate **C2** and its notable performance *in vivo* validated the use of heterobifunctional PEG linkers with a synthetic TLR7/8L for enhancing vaccine immunity. Still, the two-step bioconjugation strategy used to create conjugate **C2** had several drawbacks: (1) excess linker functionalization has been reported to sterically impair the recognition and processing of B and T cell epitopes^9,11^ and (2) cationization of proteins with low isoelectric points (pI; BSA *ca*. 4.8; CRM-197 *ca*. 5.9) due to the use of Traut’s reagent increases the risk of antigen precipitation.

Endeavoring to establish an entirely charge-neutral, PEG-based linker system that would obviate the installation of unused cross-linkers, we functionalized the moderately soluble and more challenging TLR7/8L, UM-3013, with *N*-succinimidyl *S*-acetylthioacetate (SAT(PEG)_4_), a hydrophilic PEG_4_ spacer terminating in a free thiol. By exploiting the remarkable selectivity of the thiol-maleimide Michael addition,^21^ compound **3** was generated quantitatively by adding UM-3013-SAT(PEG)_4_-SH to a stoichiometric amount of SM(PEG)_6_ in anhydrous DMF (Fig. 1B). Compound **3** proved highly soluble in PB pH 7.2 due to the hydrophilicity of the PEG_10_ linker. In addition, the terminal NHS ester enabled one-step functionalization of the lysines of CRM-197, precluding the attachment of surplus linker. This one-step bioconjugation gave rise to conjugate **C3** (Fig. 2A) whose distribution of adjuvant additions could be visualized by matrix-assisted laser desorption/ionization mass spectrometry (MALDI-TOF MS, Fig. 2B). Facile and accurate characterization of adjuvant copy # and molecular weight by number (*M*_n_) permitted on-the-fly reaction monitoring and tunable copy number addition.

As was the case with conjugate **C2**, CRM-197 conjugate **C3** induced superior antigen-specific antibody responses in mice compared to the admixed/unconjugated controls (Fig. S15, ESI†). In this experiment, two doses of the CRM-197 antigen were used (1 or 10 μg), and at both doses the use of conjugated antigen resulted in significantly greater anti-CRM-197 antibody titers as compared to using the equivalent doses of protein admixed with adjuvant. The conjugate antibody levels were even significantly higher than that achieved using a ten-fold excess of adjuvant, highlighting the capacity of antigen–adjuvant conjugation for improving the humoral immune response.

Encouraged by these results, our final task was to create a conjugate with the high potency but without the poor solubility imparted by TRL7/8L UM-3006 (**C4**, Fig. 2A). Showcasing the modularity of our new approach, a longer PEG_16_ linker was used to synthesize compound **4** (Fig. 1B), which proved highly water soluble. Compound **4** was affixed to CRM-197 in one step and in high antigen yield (73%, a 180% increase in yield compared to that of conjugate **C1**).

With our UM-3006 conjugates **C4** and **C1** in hand, we conducted head-to-head studies comparing their biological activities. The new conjugate, **C4**, proved to be similarly or somewhat more active *in vitro* than conjugate **C1** both in HEK-293 cells expressing human TLR7 (Fig. S12, ESI†) and in human peripheral blood mononuclear cells (PBMCs), producing cytokines interferon alpha (IFNα) and tumor necrosis factor alpha (TNFα; Fig. S13, ESI†). These conjugates were also tested *in vivo* by vaccinating mice with 1 or 10 μg of CRM-197 either alone, conjugated with UM-3006 (at a copy # of 4.4), or admixed with free UM-3006 at the corresponding molar ratio. Both **C4** and **C1** elicited significantly higher antibody responses than the admixed control groups, and conjugate **C4** performed similarly or slightly better than **C1** (Fig. 3). In addition, the production of antibody subtypes IgG1 and IgG2a was assessed to provide information regarding the nature of the CD4^+^ T cell responses (Fig. S16A, ESI†). Both IgG1, generally associated with a T helper 2 (Th2) response, and IgG2a, associated with a T helper 1 (Th1) response, were significantly increased by the use of either conjugate. However, conjugation favored the Th1-associated response, as the fold-increase in IgG2a was greater in the conjugate groups compared to the admixed groups (Fig. S16B, ESI†).

As a more direct measure of T cell responses, DLNs and spleen cells were collected five days following a third vaccination (for details see ESI† Section 5). T cells from mice immunized with either conjugate, **C4** or **C1**, demonstrated an increased antigen-specific release of IFNγ upon *in vitro* restimulation along with an undetectable level of IL-5 (Fig. S16C, ESI†). In contrast, antigen alone induced primarily an IL-5 response following antigen re-stimulation *in vitro*. Together, these data suggest that the conjugation of UM-3006 with an antigen results in a shift towards a Th1-dominated immune response, which has been reported to be helpful for protection against intracellular pathogens such as viruses^22,23^ and certain bacteria. Overall, these findings highlight the efficacy of co-delivering antigen and adjuvant synchronously to the same APC.

Tractable and versatile linker chemistries are of crucial importance to the advancement of conjugate vaccines. We offer a simple and modular PEG-based linker system, which minimizes the footprint of bioconjugation, facilitates characterization (crucial for accurate dosing and product development), and maximizes recovery of precious antigen during the bioconjugation process. These adjuvanted linkers are built from commercially available materials and can be used to functionalize antigenic proteins—even with extremely lipophilic TLR7/8Ls—under aqueous and non-denaturing conditions. By minimizing the number of linker attachments *via* direct NHS-mediated cross-linking, T and B cell epitope masking can be minimized. The resulting bioconjugates are remarkably immunogenic and drive robust Th1-polarized immune responses *in vivo*. The application of this technology to vaccines targeting viral and bacterial pathogens is currently underway in our lab.

This work was financially supported by the National Institute of Health and the National Institute of Allergy and Infection Disease (project # 1R01AI137146–01A1). We thank GlaxoSmithKline for the use of TLR7/8L UM-3006. We acknowledge the University of Montana Center for Translational Medicine technical teams (chemistry, immunology, and formulation) for their helpful input and assistance, especially Laura Bess and Mark Livesay for the synthesis of the TLR7/8Ls.

## Supplementary Material

Supplementary Material

## Figures and Tables

**Fig. 1:**
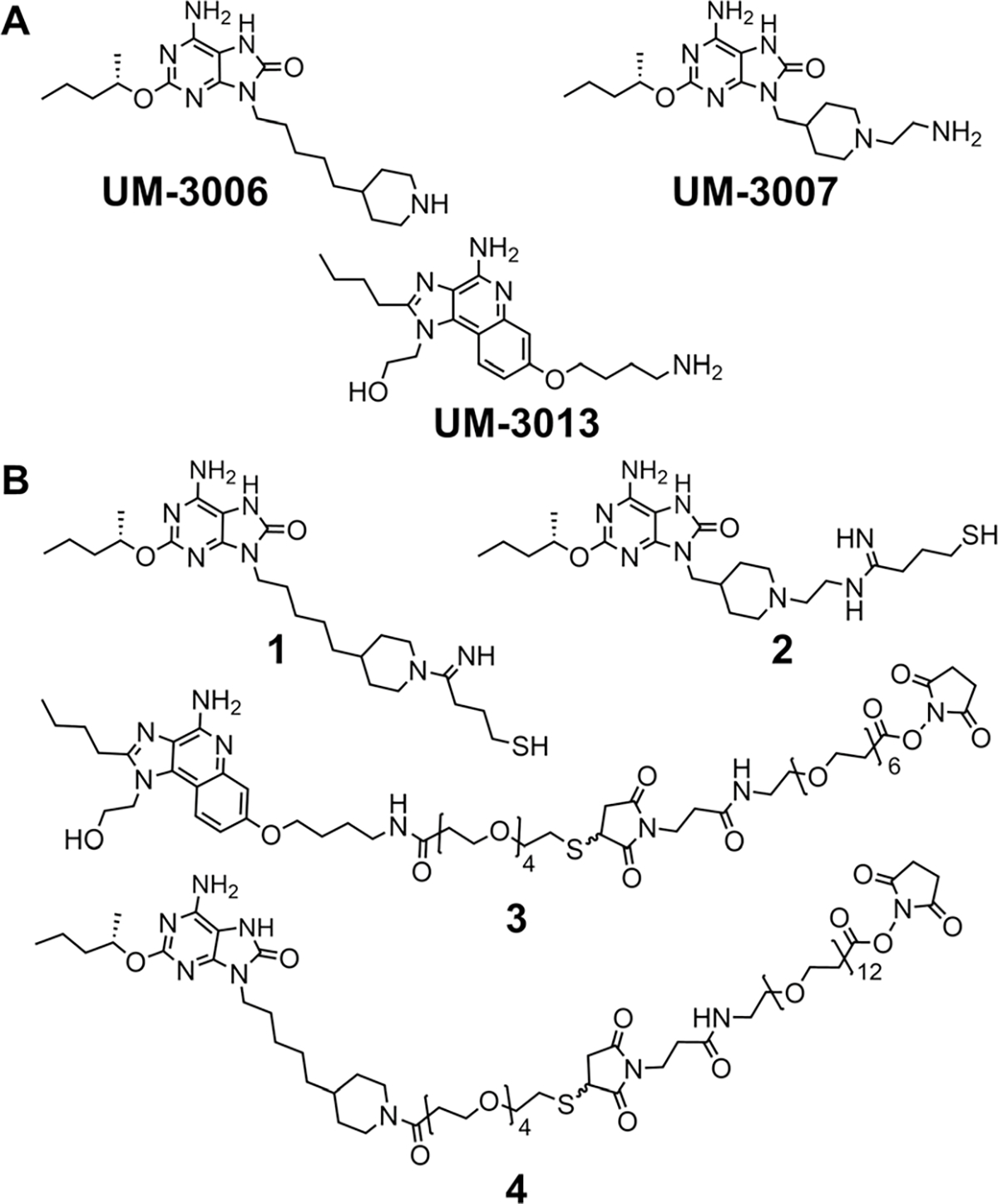
TLR7/8Ls (A) and their linker derivatizations (B). For all synthetic details including characterization, see ESI† Section 2.

**Fig. 2:**
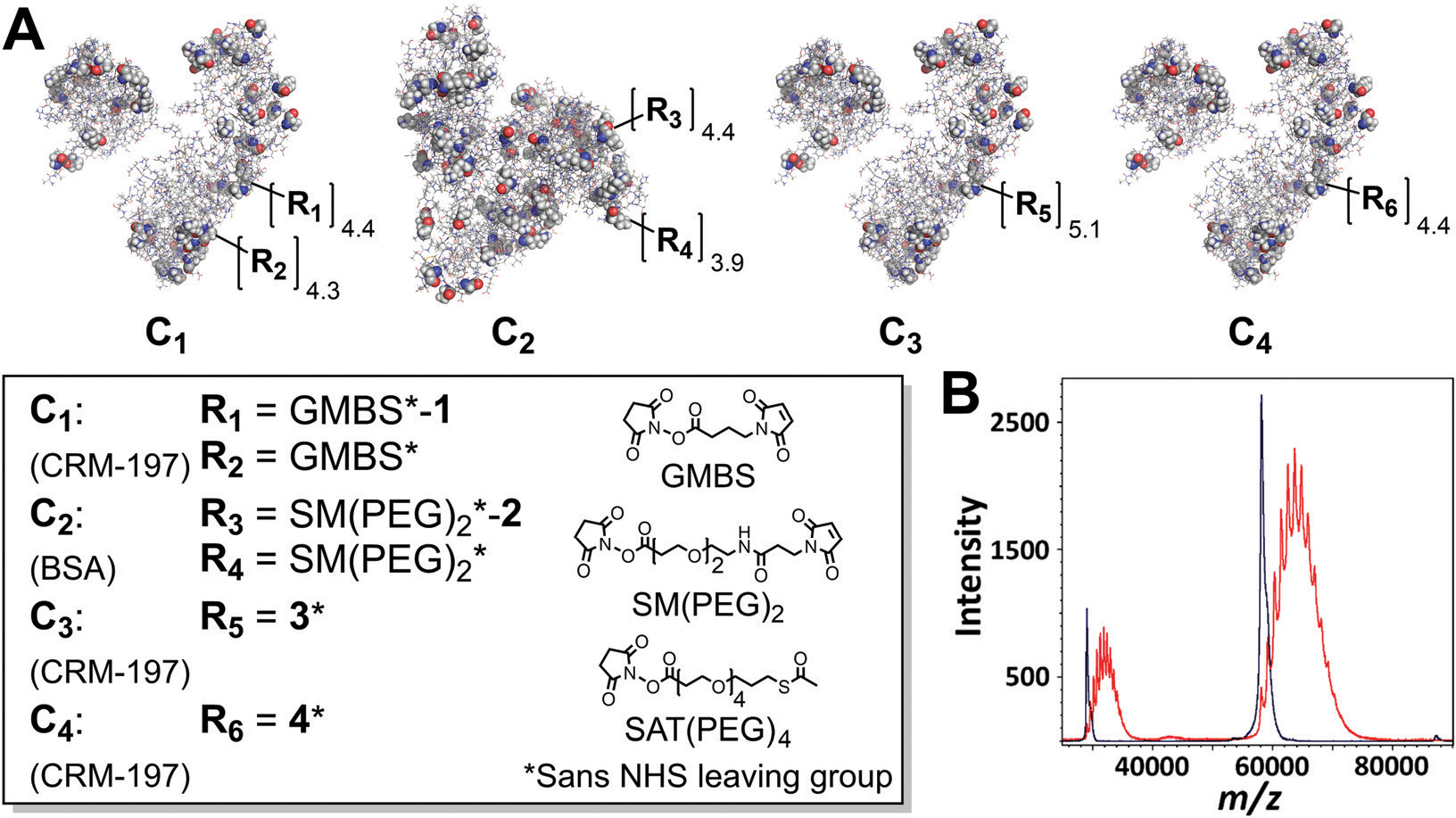
Antigen-TLR7/8L bioconjugates: (A) CRM-197 or BSA conjugates produced using two-step (**C1** and **C2**) or one-step (**C3** and **C4**) processes; "sans NHS" refers to the loss of NHS upon lysine functionalization; (B) representative MALDI-TOF mass spectra of unmodified CRM-197 (dark blue) and a bioconjugate (**C3**, red, average copy # of 5.1); [M + H]^+^ (rightmost peaks) and [M + 2H]^2+^ (leftmost peaks). For bioconjugation details including characterization, see ESI† Section 2.

**Fig. 3:**
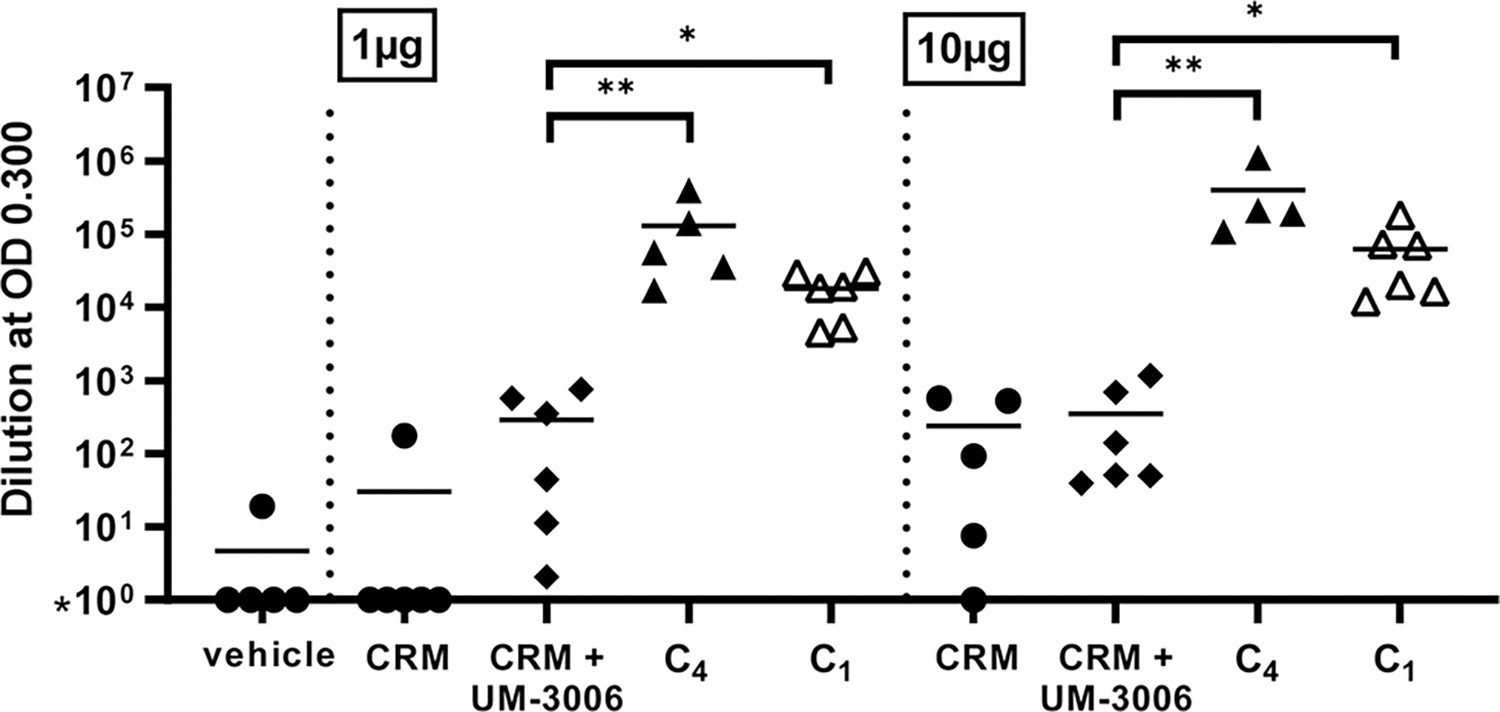
CRM-197-specific total IgG serum antibody titers 14 days after booster injection with indicated vaccine. Mice were immunized twice, 14 days apart, with 1 or 10 μg of CRM-197 either conjugated with UM-3006, admixed with the molar equivalent of free UM-3006, or alone. 14 days after secondary immunization, serum was collected, and CRM-197-specific total IgG, IgG1, and IgG2a antibody titers were measured by ELISA. For IgG isotype titers and T cell cytokine data see Fig. S16 (ESI†).

**Table 1:** Summary of antigen-TLR7/8L bioconjugate characterization data

Conjugate	Linker 1 (copy #)	Linker 2 (copy #)	Antigen recovery (%)	Unreacted antigen (%)

**C1**	GMBS* (4.3)	GMBS*-**1** (4.4)	26	2.8
**C2**	SM(PEG)_2_* (3.9)	SM(PEG)_2_*-**2** (4.4)	61	6.8
**C3**	3* (5.1)	N/A	71	2.7
**C4**	4* (4.4)	N/A	73	0.9

* Sans NHS leaving group.
