# Supplementary material for: A tractable covalent linker strategy for the production of immunogenic antigen-TLR7/8L bioconjugates
Source: Chem Commun (Camb). Author manuscript; Available in PMC 2022 May 19. (PMC9118693; doi:10.1039/d1cc00795e)
Supplement: Supplementary Material [file NIHMS1804144-supplement-Supplementary_Material.pdf]

Electronic Supplementary Material (ESI) for ChemComm.
This journal is © The Royal Society of Chemistry 2021

A Tractable Covalent Linker Strategy for the Production of Immunogenic Antigen-TLR7/8L Bioconjugates

C. J. Massena, S. K. Lathrop, C. J. Davison, R. Schoener, H. G. Bazin, J. T. Evans, and D. J. Burkhart*

Dept. of Biomedical & Pharmaceutical Sciences, University of Montana, 32 Campus Dr, Missoula, MT 59812.

Contents

Section 1: General 1

Section 2: Small-molecule synthesis and bioconjugation 5

Section 3: TLR7/8L water solubility comparison..31

Section 4: *In vitro* immunological methods and supplementary data 32

Section 5: *In vivo* immunological methods and supplementary data..36

References 42

Section 1: General

All reagents and solvents used in this study were purchased from commercial sources unless otherwise noted. See Section 2 for details on commercial sources pertaining to small-molecule synthesis and bioconjugation. See Sections 4 and 5 for details on commercial sources pertaining to immunological studies. Research-grade CRM-197 with an endotoxin level of < 1.0 EU/mL was purchased from Eurogentec. BSA was purchased from Sigma-Aldrich. TLR7/8 ligands (TLR7/8Ls) were synthesized following established procedures,[1-3] and unconjugated TLR7/8Ls were formulated in 2% *v*/*v* glycerol in water. UM-3013 (originally synthesized by Corixa Corp. following known procedures[4]) was generously provided by the Center for Translational Medicine chemistry group at the University of Montana (see Section 2 for characterization data). All reaction monitoring and HRMS were conducted using an Agilent 6520 Accurate-Mass Q-TOF LC/MS. Preparatory-scale RP-HPLC separations were carried out with a Varian PrepStar (model SD-1) equipped with a Varian ProStar UV detector and a Phenomenex Luna 10 μm Preparatory C18(2) 100 Å column (250 × 21.2 mm) using the following mobile phases and gradient (MP_A = 0.1% *v*/*v* TFA-H_2O; MP_B = CH_3CN):

Time (min)	% A	% B
0.0	97.0	3.0
2.0	97.0	3.0
20.0	3.0	97.0
25.0	3.0	97.0
27.0	97.0	3.0
28.0	97.0	3.0

Chromatograms were visualized at 280 nm. For analytical RP-HPLC, a Waters Alliance e2695 separations module equipped with a Waters 2998 PDA detector was employed using the following mobile phases and gradient (MP_A = 0.1% *v/v* TFA-H_2O; MP_B = CH_3CN):

Time (min)	% A	% B
0.0	3.0	97.0
2.0	3.0	97.0
10.0	10.0	90.0
10.5	10.0	90.0
11.0	3.0	97.0
12.0	3.0	97.0

All chromatograms were observed at 280 nm. When thorough desiccation of compounds was required, a Thermo Scientific Savant SpeedVac SPD140DDA was used. ^1H and ^{13}C NMR spectra were collected using a VNMRS Varian 500 MHz. Abbreviations for NMR multiplicities are: singlet (s), doublet (d), triplet (t), and multiplet (m). Coupling constants *J* are given in Hz. All compounds prepared for NMR were subjected to a deuterium exchange protocol to remove acidic protons (amines, amides, etc.) by dissolving the compound in deuterated MeOD followed by desiccation. To purify bioconjugates, either 30 KDa cutoff Amicon Ultra centrifugal filters (MilliporeSigma) or 7 K Zeba Spin Desalting Columns (ThermoFisher) were used (see Section 2 for details). Bioconjugate masses were determined with a Bruker microflex LRF MALDI-TOF system. MALDI-TOF samples were prepared by diluting 2 μL of sample in 2 μL of 2% *v/v* TFA-H_2O along with 2 μL of 15.2 mg/mL 2,5-dihydroxyacetophenone (DHAP, Bruker) dissolved in 75% *v/v* EtOH and aqueous 18 mg/mL diammonium hydrogen citrate (DAC, Fluka Analytics). The resulting mixture was aspirated thoroughly with a micropipette to achieve a homogenous suspension. 1 μL of the sample was pipetted onto a Bruker MSP 96 ground steel target and allowed to air dry. Copy #s were calculated by dividing the difference in masses of the two species (e.g., unmodified CRM-

197 and a bioconjugate) by the mass of the small molecule added. When a distribution of peaks corresponding to individual additions of a TLR7/8L derivative was observed, an M_n could be calculated by dividing the sum of the products of peak masses and peak intensities by the sum of the peak intensities. All unmodified protein and bioconjugate concentrations were determined using a Pierce BCA Protein Assay Kit. The endotoxin levels of all unmodified proteins and bioconjugates were determined to be < 1.0 EU/mL using a Lonza Kinetic-QCL Chromogenic LAL Assay Kit. The organic solvent during all bioconjugations was kept to < 10% *v*/*v*. Bioconjugates were subjected to sterile filtration through low-protein-binding PVDF 0.22 μm syringe filters (Millex). All buffers were prepared and sterile filtered before use. The predicted solubilities of amide-functionalized TLR7/8Ls were calculated using MarvinSketch 19.27.0. Gardiquimod was purchased from MedChemExpress as a TFA salt. For methods and supplementary data related to the immunological studies, see Sections 4 and 5.

Ligand	Antigen	Handle/linker strategy	Limitations	Ref
TLR7/8 imidazoquinoline	HIV-1 Gag; ovalbumin	• Aryl azide photoreactive handle<br>• Short aliphatic linker	• High, possibly denaturing conjugation pH (9–9.5)<br>• Non-modular linker, which would not provide additional solubility to lipophilic ligands	5-7
TLR7 imidazoquinoline	Bovine α-lactalbumin	• Isothiocyanate electrophilic handle<br>• "Zero" linker	• High, possibly denaturing conjugation pH (8.5)<br>• Non-modular linker, which would not provide additional solubility to lipophilic ligands	8
TLR7 oxoadenine	nDer p 2	• NHS ester electrophilic handle<br>• Short aliphatic linker	• Non-modular linker, which would not provide additional solubility to lipophilic ligands	9
TLR7/8 imidazoquinoline	HIV-1 gp120	• Free thiol nucleophilic handle<br>• Two-step conjugation: NHS-PEG$_8$-Maleimide addition then TLR7/8-SH addition	•Two-step conjugations that inevitably leave behind unused (and hydrolyzed) heterobifunctional linkers	10
TLR7 (unspecified)	*Streptococcus pneumoniae* RrgB	• Unspecified chemistry, but likely an aldehyde electrophilic handle<br>• $NaCNBH_3$ was used likely for imine reduction<br>• Long 71.81 Å linker (unspecified)	• High, possibly denaturing conjugation pH (8.0)<br>• Non-modular linker<br>• Reduction step involved adding low, possibly denaturing pH (4.0) buffer with $NaCNBH_3$<br>• $NaCNBH_3$ can generate free cyanide ions in solution	11
TLR7 oxoadenine	Bovine serum albumin; ovalbumin; mouse serum albumin; gastric cancer 7 antigen	• Short aliphatic linker<br>• Carboxylic acid electrophilic handle<br>• EDCI/NHS coupling chemistry	• Non-modular linker, which would not provide additional solubility to lipophilic ligands<br>• The use of EDCI can lead to protein-protein cross-linking	12, 13
TLR7 imidazoquinoline	Ovalbumin; *Plasmodium falciparum* circumsporozoite	• Extremely complex ~18 kDa polymeric, self-immolative linker	• Long, multi-step syntheses not amenable to scale up<br>• Large ~18 kDa linker constructs, which would likely not be universally compatible with every antigen	14

Table S1 Prior examples of TLR7/8-antigenic-protein bioconjugates.

Section 2: Small-molecule synthesis and bioconjugation

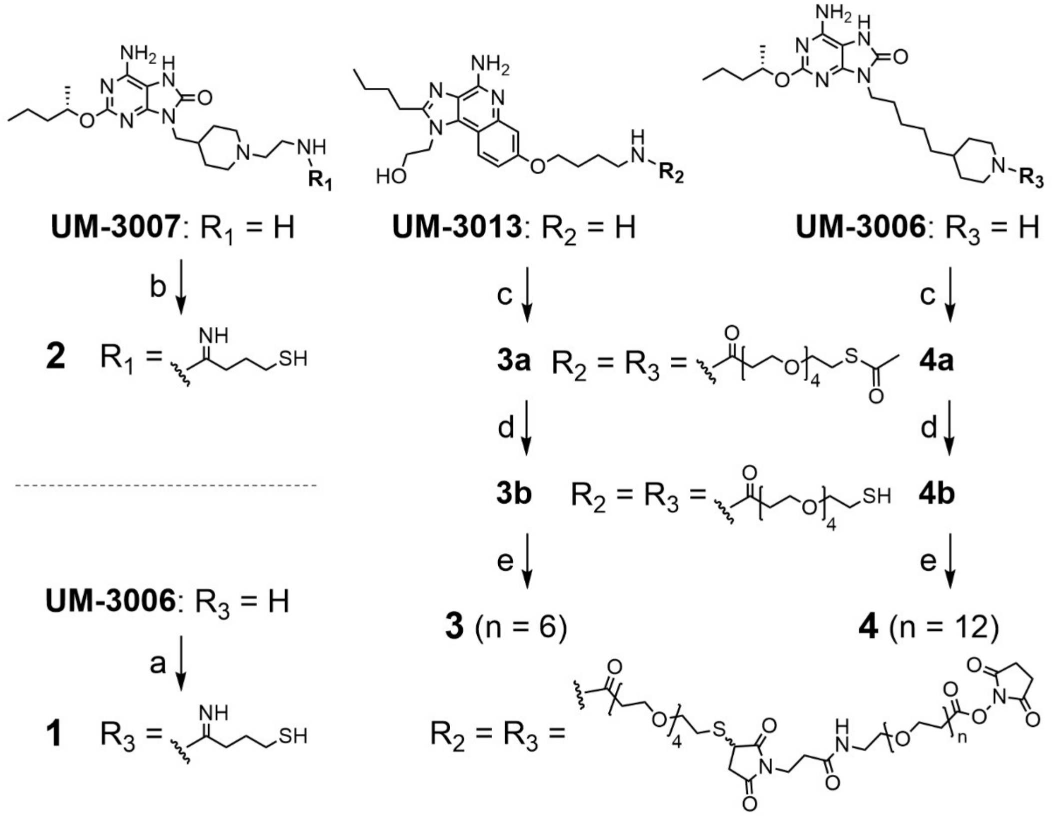

Scheme S1 Synthetic scheme of TLR7/8L derivatives: a) Traut's•HCl, DIPEA, 9:1 *v/v* DMF-H_2O, 10 min, RT, *in situ* yield not determined; b) Traut's•HCl, PB pH 7.2, 2 min, RT then 0.5% *v/v* TFA- H_2O, 85%; c) SAT$(PEG)_4$, DIPEA, DMF, 20 min, RT, quantitative (from UM-3013), 91% (from UM-3006); d) NH_2OH, PB pH 7.2, 20 min, RT, quantitative (from UM-3013), 83% (from UM-3006); e) SM$(PEG)_6$ (for **3**) or SM$(PEG)_{12}$ (for **4**), anhydrous DMF, DIPEA, 15 min, RT, quantitative.

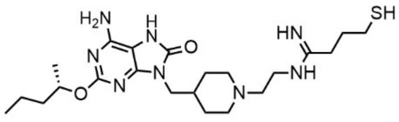

2

N-{2-[4-({6-amino-8-oxo-2-[(2S)-pentan-2-yloxy]-8,9-dihydro-7H-purin-9-yl}methyl)piperidin-1-yl]ethyl}-4-sulfanylbutanimidamide (**2**): in a glass vial, 10.2 mg (0.074 mmol, 1.0 equiv) of UM-3007•2 HCl were homogenized in 1016 μL of 0.2 M sodium phosphate buffer (NaPB) pH 7.2 with vortexing and mild sonication. In another glass vial, 98.2 mg (0.71 mmol) of Traut's reagent (2-iminothiolane•HCl, Sigma-Aldrich) were dissolved in 1963 μL of 0.2 M NaPB pH 7.2. 1723 μL of the Traut's reagent stock solution was added to the UM-3007 reaction vial while vortexing. The turbid suspension of UM-3007 turned clear and colorless. The reaction was allowed to proceed for 2 min, after which, 4261 μL of 0.5% *v/v* TFA-H_2O were added to the reaction mixture. Rapid acidification of the reaction stabilized the free thiol intermediate (**2**) for sufficient time to inject the reaction mixture onto a preparatory-scale RP-HPLC C18 column (using the equipment, mobile phases, and gradient described in Section 1). Product fractions were consolidated, and the CH_3CN was pulled off quickly using a rotoevaporator (water bath setting 50 °C). The remaining aqueous solvent was transferred to small glass vials and frozen inside a –80 °C freezer. Lyophilization overnight using a SpeedVac system gave a white powder (10.6 mg, 85%, TFA salt). Due to compound instability in solution, collection of NMR data was not feasible. HRMS ($C_{22}H_{39}N_8O_2S^+$ = $[M + H]^+$): calculated = 479.2911; found = 479.2928.

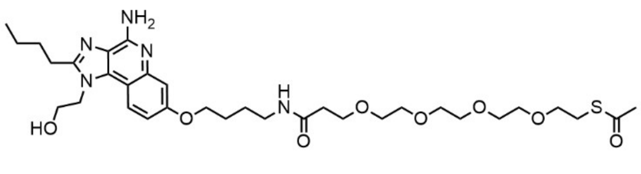

3a

N-(4-{[4-amino-2-butyl-1-(2-hydroxyethyl)-1H-imidazo[4,5-c]quinolin-7-yl]oxy}butyl)-2-oxo-6,9,12,15-tetraoxa-3-thiaoctadecan-18-amide (**3a**): in a plastic vial, 20.2 mg (0.050 mmol, 1.0 equiv) of UM-3013•HCl were dissolved in 4048 μL of DMSO to achieve a 5.0 mg/mL solution. Separately, a 50.0 mg/mL stock solution of SAT(PEG)$_4$ (PEGylated *N*-succinimidyl *S*-acetylthioacetate, ThermoFisher) in anhydrous DMF was prepared. Note: when prepared in anhydrous DMF, this stock solution is stable at –20 °C for months. 627 μL (0.074 mmol, 1.5 equiv) of the SAT(PEG)$_4$ stock solution were transferred to the adjuvant vial along with 13.0 μL (1.5 equiv) of DIPEA to initiate the reaction; the reaction mixture was then vortexed. The reaction was allowed to sit without stirring at RT for 20 min. LC/MS was used to monitor the reaction and confirm completion. The target compound was purified by preparatory-scale RP-HPLC (using the equipment, mobile phases, and gradient described in Section 1) to afford a clear oil (41.3 mg, quantitative). On account of the acetyl protecting group on the terminal thiol, compound **3a** exhibited excellent stability and could be subjected to standard desiccation (rotoevaporation followed by Speed-Vac drying). ^{1}H NMR (500 MHz, CD_3OD) δ 8.02 (d, *J* = 9.1 Hz, 1H), 7.16 (d, *J* = 2.5 Hz, 1H), 7.01 (dd, *J* = 9.1, 2.6 Hz, 1H), 4.66 (t, *J* = 5.5 Hz, 2H), 4.12 (t, *J* = 6.3 Hz, 2H), 4.00 (t, *J* = 5.5 Hz, 2H), 3.72 (t, *J* = 6.1 Hz, 2H), 3.61–3.57 (m, 10 H), 3.56–3.54 (m, 4H), 3.52 (t, *J* = 6.5 Hz, 2H), 3.29 (t, *J* = 7.0, 2H, partially overlapping with residual solvent peak), 3.06–3.00 (m, 4H), 2.44 (t, *J* = 6.1 Hz, 2H), 2.29 (s, 3H), 1.94–1.85 (m, 4H), 1.77–1.70 (m, 2H), 1.58–1.50 (m, 2H), 1.04 (t, *J* = 7.4 Hz, 3H). ^{13}C NMR (126 MHz, CD_3OD) δ 197.01, 174.03, 160.13, 156.28, 152.62, 135.80,

125.40, 122.80, 114.46, 112.29, 110.08, 107.83, 71.58, 71.55, 71.50, 71.47, 71.37, 71.29, 70.78, 68.80, 68.35, 61.37, 40.10, 37.81, 30.80, 30.42, 29.60, 27.98, 27.70, 27.18, 23.59, 14.18. HRMS ($C_{33}H_{52}N_5O_8S^+$ = $[M + H]^+$): calculated = 678.3531; found = 678.3534.

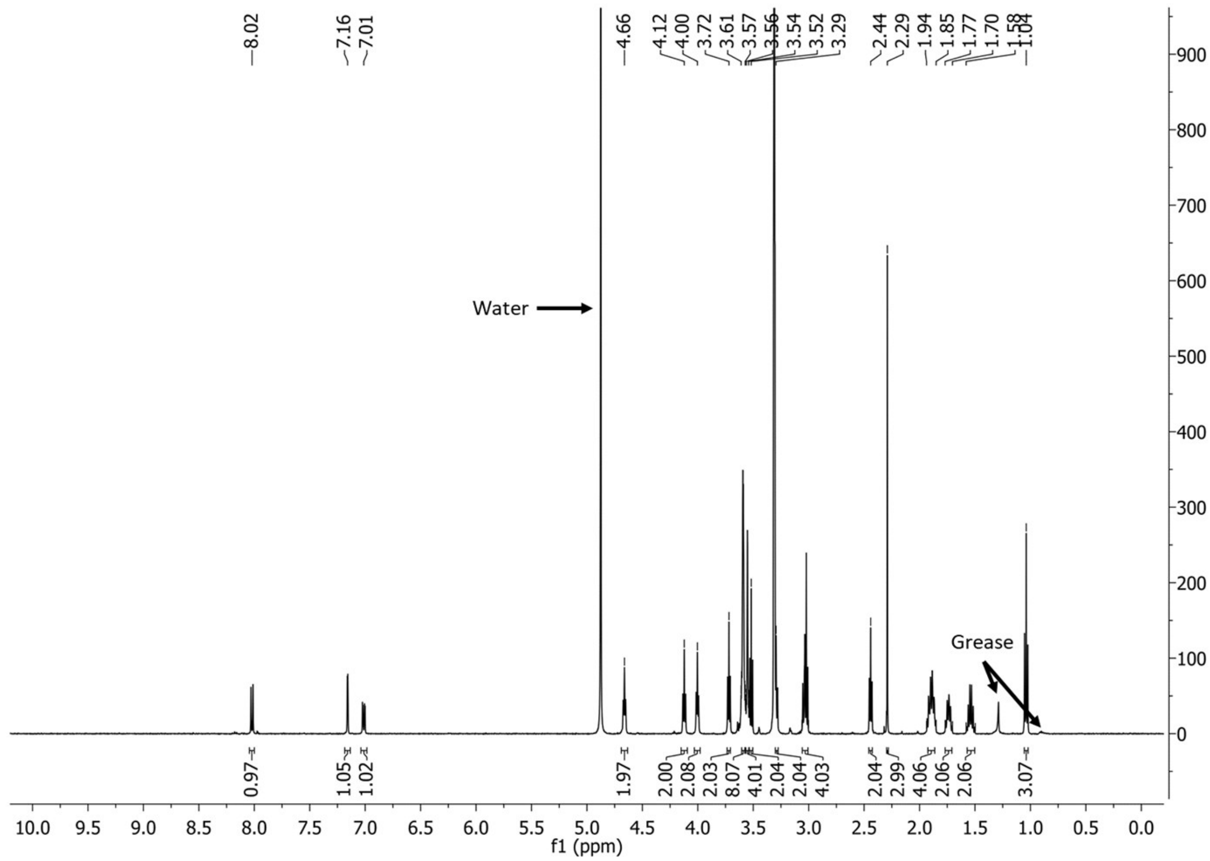

Fig. S1 ^{1}H NMR spectrum of compound **3a** (500 MHz, CD_3OD, 298 K).

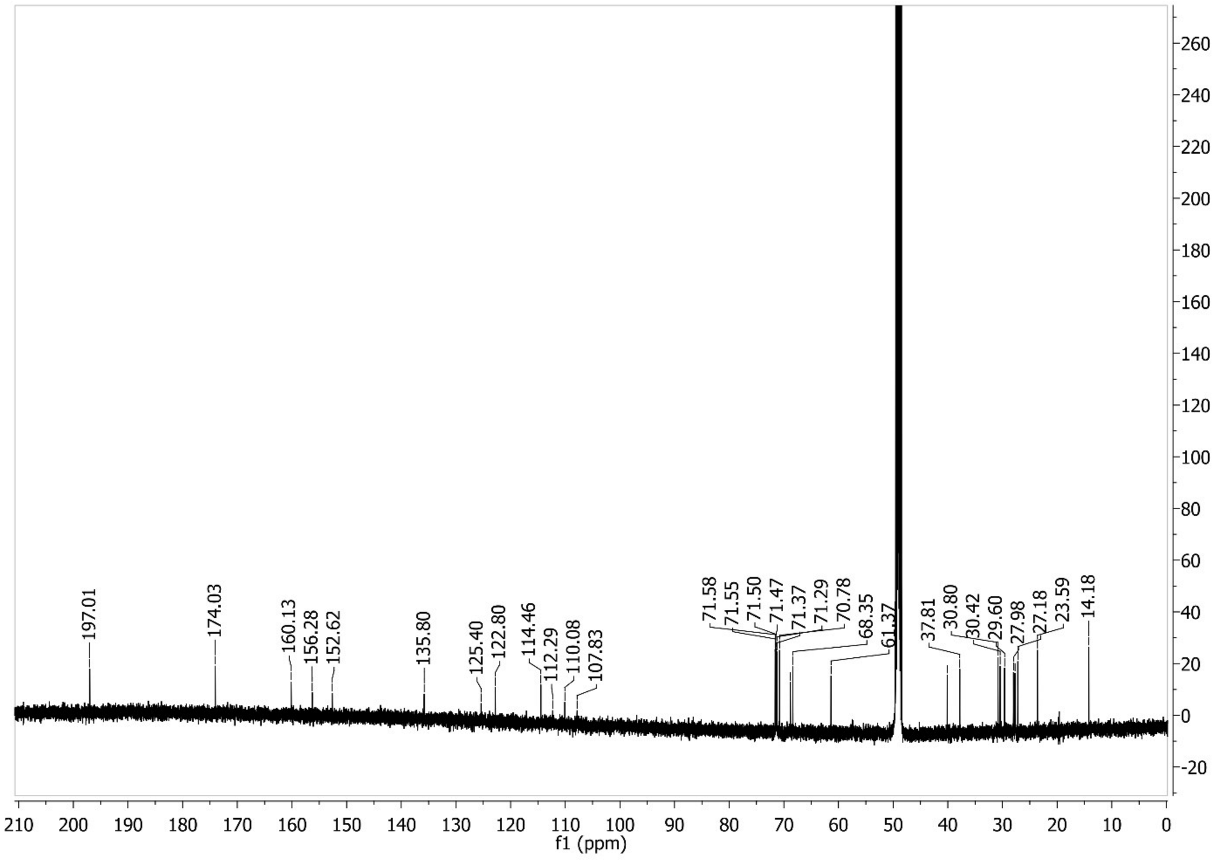

Fig. S2 ^{13}C NMR spectrum of compound **3a** (126 MHz, CD_3OD, 298 K).

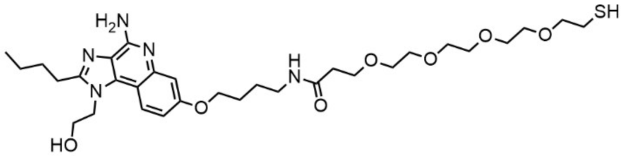

3b

N-(4-{[4-amino-2-butyl-1-(2-hydroxyethyl)-1H-imidazo[4,5-c]quinolin-7-yl]oxy}butyl)-1-sulfanyl-3,6,9,12-tetraoxapentadecan-15-amide (**3b**): in a plastic vial, a 1.0 M NH_2OH•HCl stock solution was prepared with 0.2 M NaPB pH 7.2 (pH adjustment with 10 M NaOH was required). Note: for best results, the NH_2OH solution should be prepared immediately before use. In a separate plastic vial, 40.0 mg of **3a** were dissolved in 4000 μL of DMF to achieve a 10.0 mg/mL

solution. While vortexing, 4000 μL of the NH_2OH stock solution were added to **3a**. The exothermic reaction was allowed to proceed for 20 min with vigorous shaking but without heating or cooling, during which time turbidity was observed. LC/MS analysis confirmed complete deacetylation of **3a**. The entirety of the reaction solution was injected onto a preparatory-scale RP-HPLC C18 column (using the equipment, mobile phases, and gradient described in Section 1), and the product fractions were consolidated. The CH_3CN was pulled off quickly using a rotoevaporator (water bath setting 50 °C). The remaining aqueous solvent was transferred to small glass vials and frozen inside a –80 °C freezer. Lyophilization overnight using a SpeedVac system gave a clear oil (38.1 mg, quantitative), which was used as quickly as possible due to its susceptibility to oxidation (disulfide formation). Note: compound **3b** can be stored temporarily at –20 °C within a secondary container filled with Drierite. In view of the instability of **3b**, two strategies can be employed: 1) stockpile **3a**, which is very stable, and deacetylate as needed or 2) synthesize **3b** via a one-pot reaction, i.e. complete preps for **3a** and **3b** sans purification of **3a**. To execute the latter, care must be taken to use a stoichiometric amount of SAT(PEG)$_4$, which can be accomplished by titrating UM-3013 with a stock solution of SAT(PEG)$_4$ until reaction completion is achieved (monitored by LC/MS). Due to compound instability in solution, collection of NMR data was not feasible. HRMS ($C_{31}H_{50}N_5O_7S^+$ = $[M + H]^+$): calculated = 636.3425; found = 636.3393.

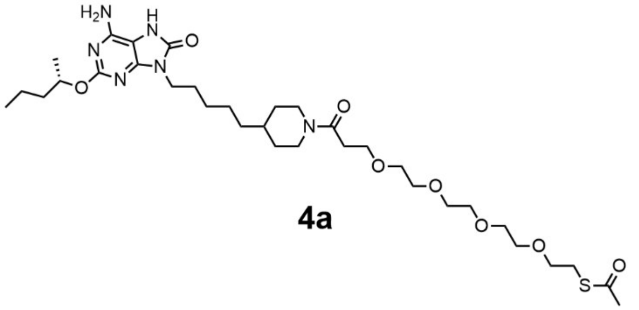

4a

18-[4-(5-{6-amino-8-oxo-2-[(2S)-pentan-2-yloxy]-8,9-dihydro-7H-purin-9-yl}pentyl)piperidin-1-yl]-6,9,12,15-tetraoxa-3-thiaoctadecane-2,18-dione (**4a**): in a plastic vial, 50.6 mg (0.13 mmol, 1.0 equiv) of UM-3006•HCl were dissolved in 2024 μL of DMSO. Separately, a 100.0 mg/mL stock solution of SAT(PEG)$_4$ (PEGylated *N*-succinimidyl *S*-acetylthioacetate, ThermoFisher) in anhydrous DMF was prepared. Note: when prepared in anhydrous DMF, the stock solution is stable at –20 °C for months. 546 μL (0.13 mmol, 1.0 equiv) of the SAT(PEG)$_4$ stock solution were added to the adjuvant vial. 33.9 μL (1.5 equiv) of DIPEA were added to the same vial (which was vortexed) to initiate the reaction. The reaction was allowed to proceed for 20 min at RT without stirring. LC/MS analysis confirmed complete addition of SAT(PEG)$_4$ to UM-3006. The target compound was purified by preparatory-scale RP-HPLC (using the equipment, mobile phases, and gradient described in Section 1) to afford a clear oil (95.3 mg, 91%). On account of the acetyl protecting group on the terminal thiol, compound **4a** exhibited excellent stability and could be subjected to standard desiccation (rotoevaporation followed by Speed-Vac drying). ^{1}H NMR (500 MHz, CD_3OD) δ 5.21–5.14 (m, 1H), 4.49 (d, *J* = 13.4 Hz, 1H), 3.99 (d, *J* = 13.8 Hz, 1H), 3.83 (t, *J* = 7.0 Hz, 2H), 3.73 (dd, *J* = 9.8, 3.4 Hz, 2H), 3.63–3.59 (m, 12H), 3.57 (t, *J* = 6.5 Hz, 2H), 3.09–3.00 (m, 3H), 2.72–2.54 (qdd, *J* = 15.5, 14.0, 4.5 Hz, 3H), 2.31 (s, 3H), 1.80–1.68 (m, 5H), 1.63–1.55 (m, 1H), 1.53–1.31 (m, 10H), 1.29–1.22 (m, 2H), 1.12 (qd, *J* = 12.5, 4.1 Hz, 1H), 1.06–0.99 (m, 1H), 0.96 (t, *J* = 7.4 Hz, 3H). ^{13}C NMR (126 MHz, CD_3OD) δ 196.97, 171.69, 160.82, 155.02, 151.20,

99.50, 74.83, 71.62, 71.56, 71.56, 71.55, 71.49, 71.35, 70.82, 68.57, 47.55, 43.38, 41.00, 39.46, 37.28, 37.05, 34.50, 33.97, 33.18, 30.44, 29.65, 29.24, 27.78, 27.15, 20.27, 19.77, 14.36. HRMS ($C_{33}H_{57}N_6O_8S^+$ = $[M + H]^+$): calculated = 697.3953; found = 697.3956.

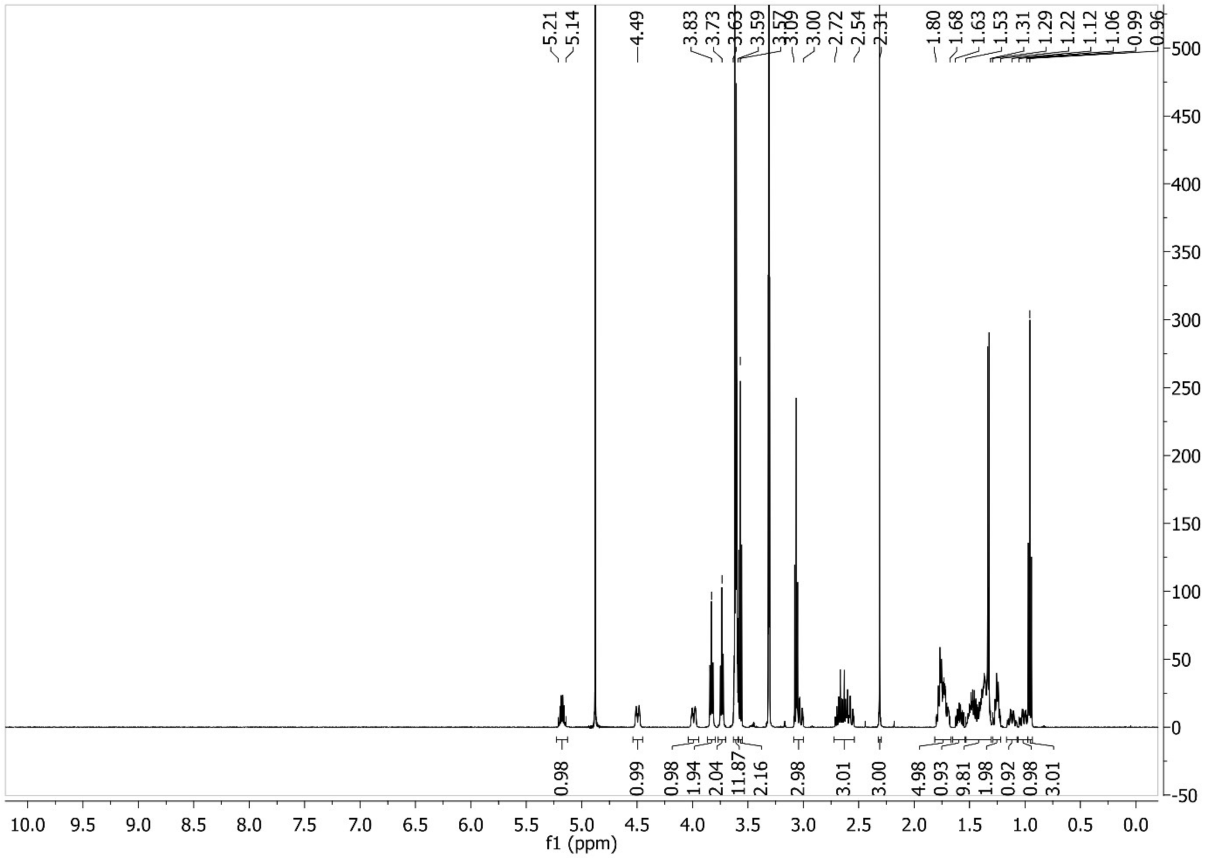

Fig. S3 ^1H NMR spectrum of compound **4a** (500 MHz, CD_3OD, 298 K).

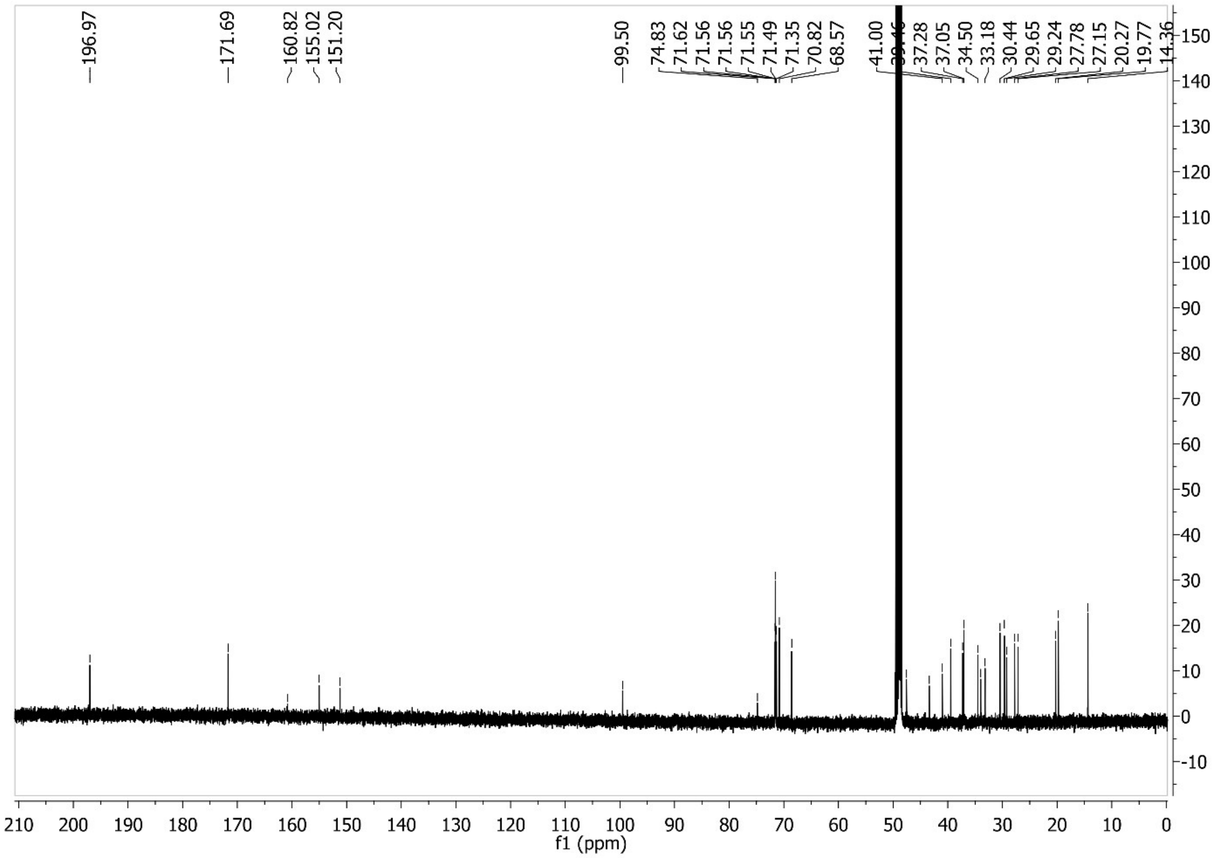

Fig. S4 ^{13}C NMR spectrum of compound **4a** (126 MHz, CD_3OD, 298 K).

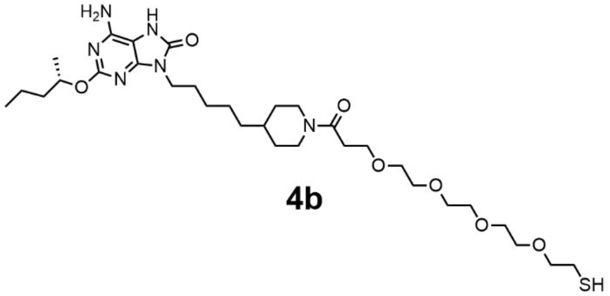

6-amino-2-[(2S)-pentan-2-yloxy]-9-{5-[1-(1-sulfanyl-3,6,9,12-tetraoxapentadecanoyl)piperidin-4-yl]pentyl}-8,9-dihydro-7H-purin-8-one (**4b**): in a plastic vial, a 1.0 M NH_2OH•HCl stock solution was prepared with 0.2 M NaPB pH 7.2 (pH adjustment with 10 M NaOH was required). Note: for best results, the NH_2OH solution should be prepared immediately before use. In a

separate plastic vial, 9.1 mg of **4a** were dissolved in 910 μL of DMF to achieve a 10.0 mg/mL solution. While vortexing, 908 μL of the NH_2OH stock solution were added to **4a**. The exothermic reaction was allowed to proceed for 20 min with vigorous shaking but without heating or cooling, during which time turbidity was observed. LC/MS analysis confirmed complete deacetylation of **4a**. The entirety of the reaction solution was injected onto a preparatory-scale RP-HPLC C18 column (using the equipment, mobile phases, and gradient described in Section 1), and the product fractions were consolidated. The CH_3CN was pulled off quickly using a rotoevaporator (water bath setting 50 °C). The remaining aqueous solvent was transferred to small glass vials and frozen inside a –80 °C freezer. Lyophilization overnight using a SpeedVac system gave a clear oil (6.1 mg, 83%), which was used as quickly as possible due to its susceptibility to oxidation (disulfide formation). Note: compound **4a** can be stored temporarily at –20 °C within a secondary container filled with Drierite. In view of the instability of this intermediate, two strategies can be employed: 1) stockpile **4a**, which is very stable, and deacetylate as needed or 2) synthesize **4b** via a one-pot reaction, i.e. complete preps for **4a** and **4b** sans purification of **4a**. To execute the latter, care must be taken to use a stoichiometric amount of $SAT(PEG)_4$, which can be accomplished by titrating UM-3006 with a stock solution of $SAT(PEG)_4$ until reaction completion is achieved (monitored by LC/MS). Due to compound instability in solution, collection of NMR data was not feasible. HRMS ($C_{31}H_{55}N_6O_7S^+$ = $[M + H]^+$): calculated = 655.3847; found = 655.3872.

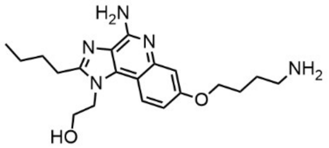

UM-3013

2-[4-amino-7-(4-aminobutoxy)-2-butyl-1H-imidazo[4,5-c]quinolin-1-yl]ethan-1-ol (UM-3013): originally synthesized by Corixa Corp. following known procedures,[4] this compound was generously provided by the Center for Translational Medicine chemistry group at the University of Montana. ^{1}H NMR (400 MHz, CD_3OD) δ 8.22 (d, 1H), 7.31 (d, 1H), 7.20 (dd, 1H), 4.71 (t, 2H), 4.20 (t, 2H), 4.01 (t, 2H), 3.05 (m, 4H), 1.94 (m, 6H), 1.55 (q, 2H), 1.05 (t, 3H). ^{13}C NMR (126 MHz, CD_3OD) 161.4, 159.0, 150.1, 138.0, 137.2, 124.7, 123.8, 116.2, 108.4, 102.8, 69.1, 61.3, 49.3, 40.6, 30.2, 28.0, 27.2, 25.6, 23.5, 14.2. ESI-MS ($C_{20}H_{30}N_5O_2^+$ = $[M + H]^+$): calculated = 372.24; found = 372.30.

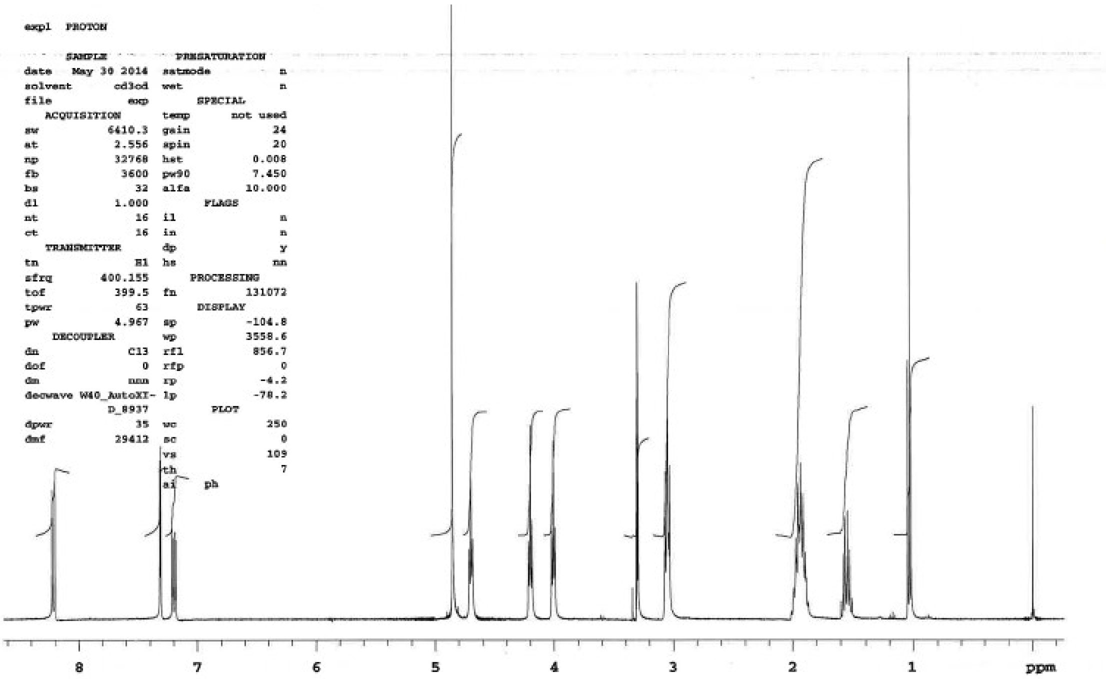

exp1 PROTON
SAMPLE
date May 30 2014
solvent cd3od
file exp
ACQUISITION
sw 6410.3
at 2.556
np 32768
fb 3600
bs 32
d1 1.000
nt 16
ct 16
TRANSMITTER
tn H1
sfrq 400.155
tof 399.5
tpwr 63
pw 4.967
DECOUPLER
dn C13
dof 0
dm nnn
decwave W40_AutoXI-D_8937
dpwr 35
dmf 29412
PRESATURATION
satmode n
wet n
SPECIAL
temp not used
gain 24
spin 20
hst 0.008
pw90 7.450
alfa 10.000
FLAGS
il n
in n
dp y
hs nn
PROCESSING
fn 131072
DISPLAY
sp -104.8
wp 3558.6
rfl 856.7
rfp 0
rp -4.2
lp -78.2
PLOT
wc 250
sc 0
vs 109
th 7
ai ph
8
7
6
5
4
3
2
1
ppm

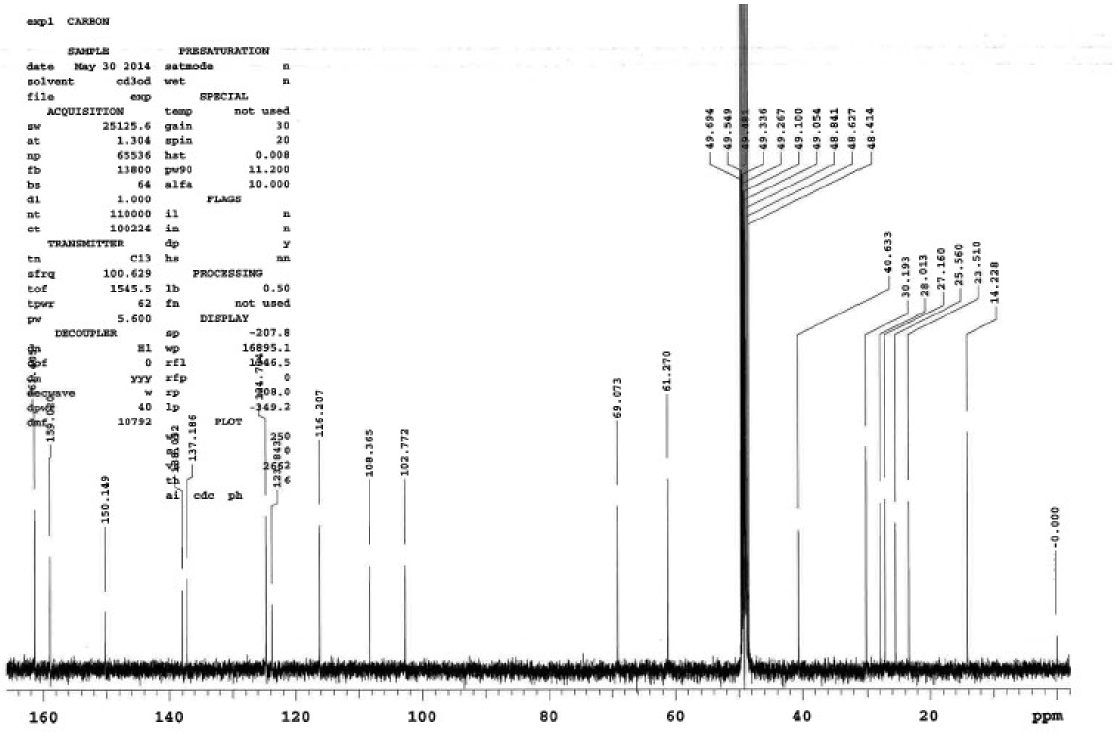

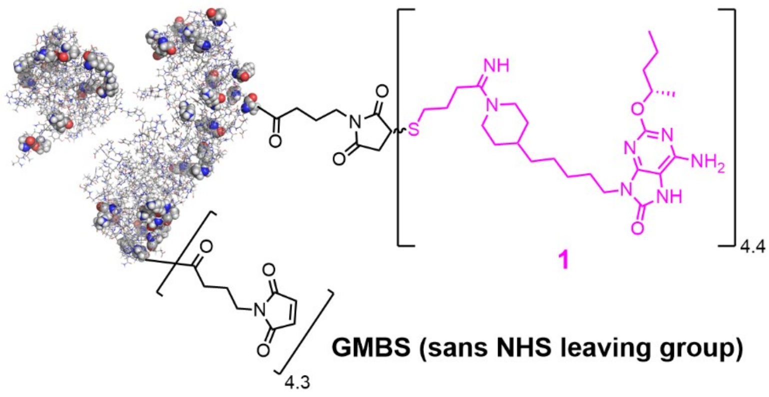

C_1: CRM-197-GMBS-1 (with unused GMBS groups)

Conjugate $\mathbf{C_1}$ was synthesized in three parts: a) CRM-197 was functionalized with 8.7 copies of GMBS (sans NHS leaving group), b) compound **1** was synthesized separately and *in situ* by reacting UM-3006 with Traut's reagent, and c) post-purification, the GMBS-functionalized CRM-197 was overlaid with compound **1** to achieve an adjuvant copy # of 4.4. Note: unlike compound **2**, compound **1** cannot be isolated.

Part a: in a plastic conical vial, a 0.5 mg/mL solution of 6.0 mg of CRM-197 (0.00010 mmol) in 10 mM PB pH 7.2 was prepared from a more concentrated stock solution of CRM-197. In another plastic vial, 3.0 mg (0.011 mmol) of GMBS (*N*-γ-maleimidobutyryl-oxysuccinimide ester, Interchim Inc.) were dissolved in 300 μL of DMSO to create a 10.0 mg/mL stock solution. 245 μL (0.0087 mmol) of the GMBS stock solution were added slowly to the protein vial while vortexing gently. The reaction appeared clear and colorless and was allowed to proceed for 30 min at RT without stirring. A 15 mL Amicon Ultra centrifugal filter (30 KDa cutoff) was used to purify the GMBS-functionalized CRM-197 (a total of four spins), and a final protein concentration of roughly 0.5 mg/mL was achieved. The copy # of GMBS moieties was characterized by MALDI-TOF MS (8.7 copies). The final product was used immediately after preparation.

Part b: in a plastic vial, 5.4 mg (0.013 mmol, 1.0 equiv) of UM-3006 were dissolved in 217 μL of 9:1 *v*/*v* DMF-H_2O to create a 25.0 mg/mL solution. Separately, 2.1 mg (0.015 mmol) of Traut's reagent (2-iminothiolane•HCl, Sigma-Aldrich) were dissolved in 83.2 μL of 9:1 *v*/*v* DMF-H_2O to create a 25.0 mg/mL solution. The entirety of the Traut's reagent stock solution was added to the adjuvant vial. Then 4.1 μL (1.9 equiv) of DIPEA were added to the reaction, which was vortexed and allowed to react for 10 min at RT without stirring. Compound **1** was used immediately after preparation due to its instability.

Part c: 120.0 μL of the stock solution of **1** were added to the GMBS-functionalized CRM-197 from Part a while vortexing gently. The solution looked clear and colorless, and the reaction was allowed to proceed without stirring at RT for 40 min. A 15 mL Amicon Ultra centrifugal filter (30 KDa cutoff) was used to purify the conjugate against 10 mM PB pH 7.2 for a total of four spins. Note: long spin times were required likely on account of protein scaling on the filter. The purified protein was sterile filtered. MALDI-TOF MS was used to determine an adjuvant copy # of 4.4. A BCA assay kit was utilized to determine the total mass and yield of conjugate $\mathbf{C_1}$ (1.7 mg, 26%).

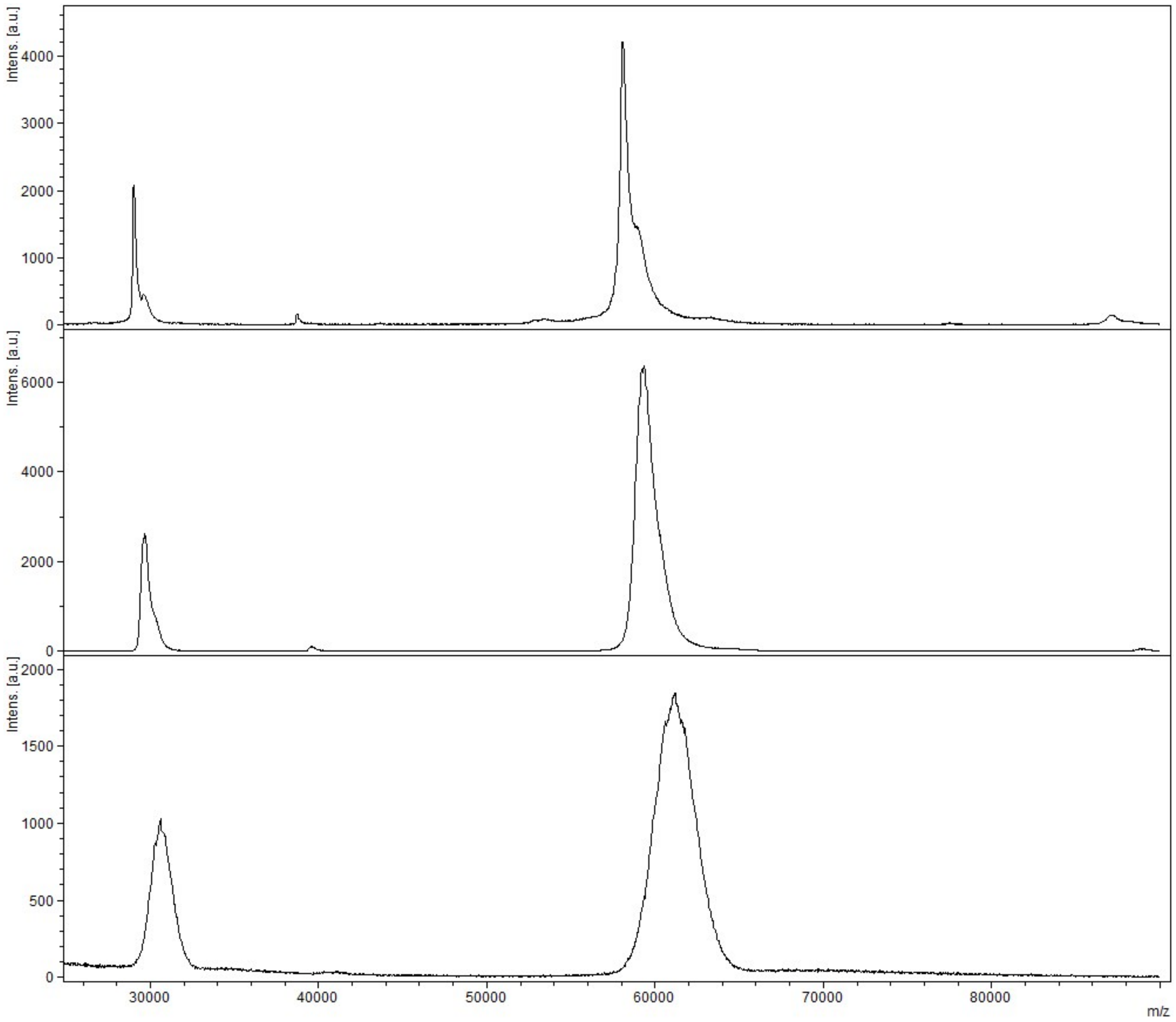

Fig. S5 MALDI-TOF mass spectrum of unmodified CRM-197, GMBS-functionalized CRM-197, and conjugate $\mathbf{C_1}$ (top to bottom). Rightmost peaks correspond to $[M + H]^+$ ions; leftmost peaks correspond to $[M + 2H]^{2+}$ ions.

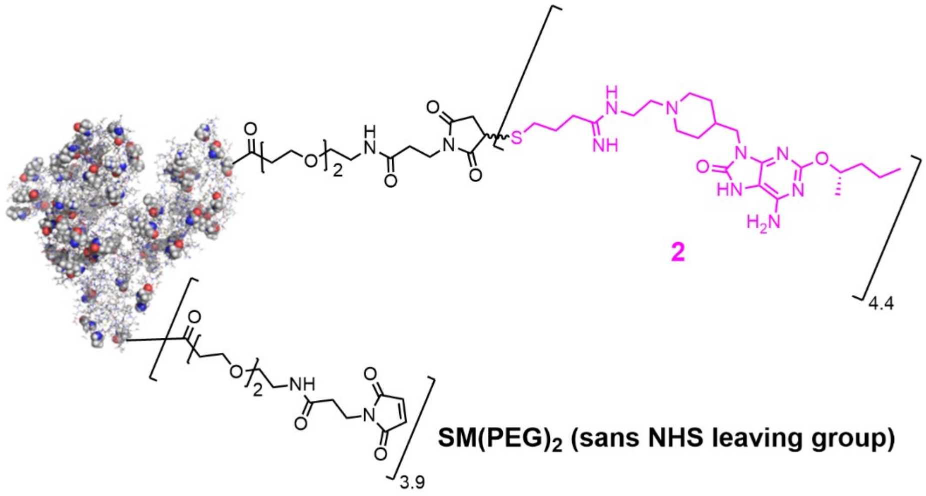

C_2: BSA-SM(PEG)$_2$-2 (with unused SM(PEG)$_2$ groups)

Conjugate **C_2** was synthesized in two parts: a) BSA was functionalized with 8.3 copies of SM(PEG)$_2$ (sans NHS leaving group) and b) post-purification, the SM(PEG)$_2$-functionalized BSA was overlaid with compound **2** to achieve a UM-3007 copy # of 4.4.

Part a: in a plastic conical vial, a 2.0 mg/mL solution of 8.0 mg of BSA (0.00012 mmol, Sigma-Aldrich) in 10 mM PB pH 7.2 was prepared and sterile filtered. Separately, a 20.0 mg/mL SM(PEG)$_2$ (succinimidyl-[(*N*-maleimidopropionamido)-diethyleneglycol] ester, ThermoFisher) stock solution was prepared in DMSO. 61.5 μL (0.0029 mmol) of the SM(PEG)$_2$ stock solution were added slowly to the protein solution while vortexing gently. The reaction appeared clear and colorless and was allowed to proceed for 30 min at RT without stirring. A 15 mL Amicon Ultra centrifugal filter (30 KDa cutoff) was used to purify the SM(PEG)$_2$-functionalized BSA (a total of four spins), and a final concentration of roughly 1.1 mg/mL was achieved. The copy # of SM(PEG)$_2$ moieties was characterized by MALDI-TOF MS (8.3 copies). The maleimide-functionalized BSA was used immediately.

Part b: in a plastic vial, 0.78 mg (0.0013 mmol) of **2**•TFA were dissolved in 62.3 μL of DMSO, resulting in a 12.5 mg/mL stock solution. Separately, 1500 μL (1.7 mg, 0.000024 mmol) of SM$(PEG)_2$-functionalized BSA were pipetted into a fresh plastic vial. 38.5 μL (0.48 mg, 0.00081 mmol) of the stock solution of **2** were added to the protein vial while vortexing gently. The solution looked clear and colorless, and the reaction was allowed to proceed without stirring at RT for 12 h. A 4 mL Amicon Ultra centrifugal filter (30 KDa cutoff) was used to purify the conjugate against 10 mM PB pH 7.2 for a total of four spins. MALDI-TOF MS was used to determine an adjuvant copy # of 4.4. The clear and colorless formulation was sterile filtered. A BCA assay kit was utilized to determine the mass and yield of conjugate $\mathbf{C_2}$ (1.0 mg, 61%).

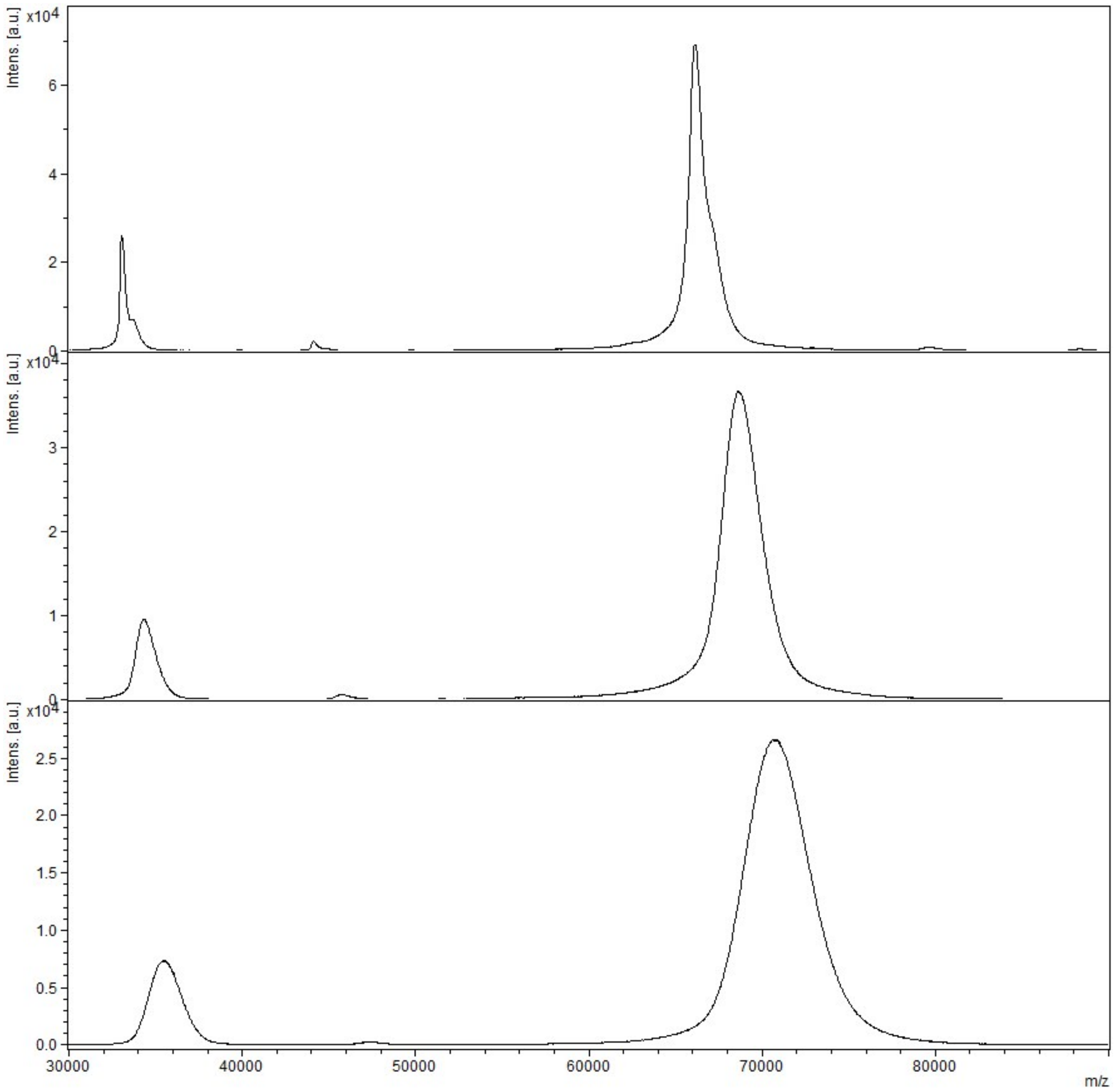

Fig. S6 MALDI-TOF mass spectrum of unmodified BSA, SM(PEG)$_2$-functionalized BSA, and conjugate $\mathbf{C_2}$ (top to bottom). Rightmost peaks correspond to $[M + H]^+$ ions; leftmost peaks correspond to $[M + 2H]^{2+}$ ions.

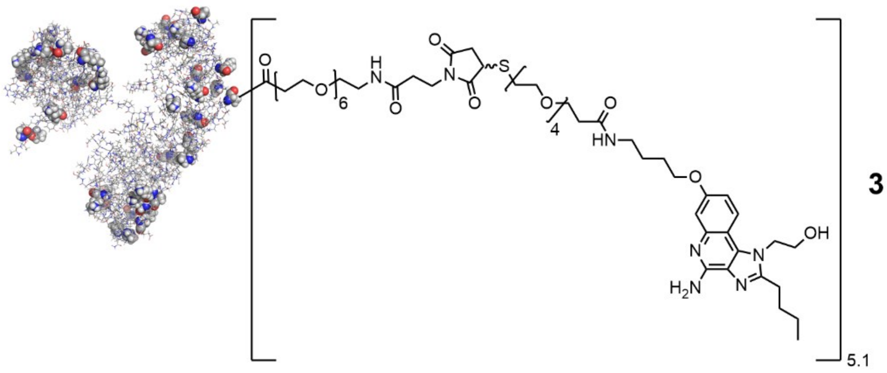

3 sans NHS leaving group

C_3: CRM-197-3

Conjugate **C_3** was synthesized in two parts: a) compound **3** was synthesized from **3b** *in situ* and b) the stock solution of compound **3** was added to CRM-197 to create conjugate **C_3**.

Part a: compound **3** was created *in situ* and used immediately after preparation. In a plastic vial, 4.7 mg (0.0079 mmol, 1.0 equiv) of SM(PEG)$_6$ (succinimidyl-[(*N*-maleimidopropionamido)-hexaethyleneglycol] ester, ThermoFisher) were dissolved in 237 μL of anhydrous DMF. In a separate plastic vial, a 10.0 mg/mL solution of **3b** was prepared by dissolving 11.4 mg of the compound in 1135 μL of anhydrous DMF. The SM(PEG)$_6$ solution was titrated with **3b** and monitored by LC/MS. This was accomplished by adding a total of 766 μL (7.7 mg, 0.010 mmol) of the **3b** stock solution to the SM(PEG)$_6$ stock solution in the presence of 4.1 μL (3.0 equiv) of DIPEA. After each addition of compound **3b**, the reaction was allowed to proceed at RT for 15 min without stirring after some initial vortexing. This titration resulted in the full *in situ* conversion of the SM(PEG)$_6$ starting material to **3** (quantitative). Although used immediately after preparation, the stock solution of **3** exhibited good stability for weeks at –20 °C. HRMS ($C_{57}H_{89}N_8O_{20}S^+$ = $[M + H]^+$): calculated = 1237.5908; found = 1237.5935.

Part b: in a plastic conical vial, a 1.1 mg/mL solution of 14.1 mg of CRM-197 (0.00024 mmol) in 10 mM PB pH 7.2 was prepared from a more concentrated stock solution of CRM-197.

While vortexing gently, 290 μL (0.0030 mmol) of the stock solution of **3** were slowly added to the protein vial. The reaction appeared clear and colorless and was allowed to proceed at RT without stirring for 45 min. Conveniently, the reaction progress could be monitored by MALDI-TOF MS. A 15 mL Amicon Ultra centrifugal filter (30 KDa cutoff) was used to purify the conjugate against 10 mM PB pH 7.2 for a total of six spins. Due to the relatively large molecular weight of the TLR7/8L derivative, the elimination of free, unconjugated compound **3** was confirmed with analytical RP-HPLC (using the equipment, mobile phases, and gradient described in Section 1). MALDI-TOF MS was used to determine an adjuvant copy # of 5.1. The clear and colorless formulation was sterile filtered. A BCA assay kit was utilized to determine the total mass and yield of conjugate **C_3** (10.9 mg, 71%). This protocol was amenable to scale up (a 28 mg conjugation was successfully completed).

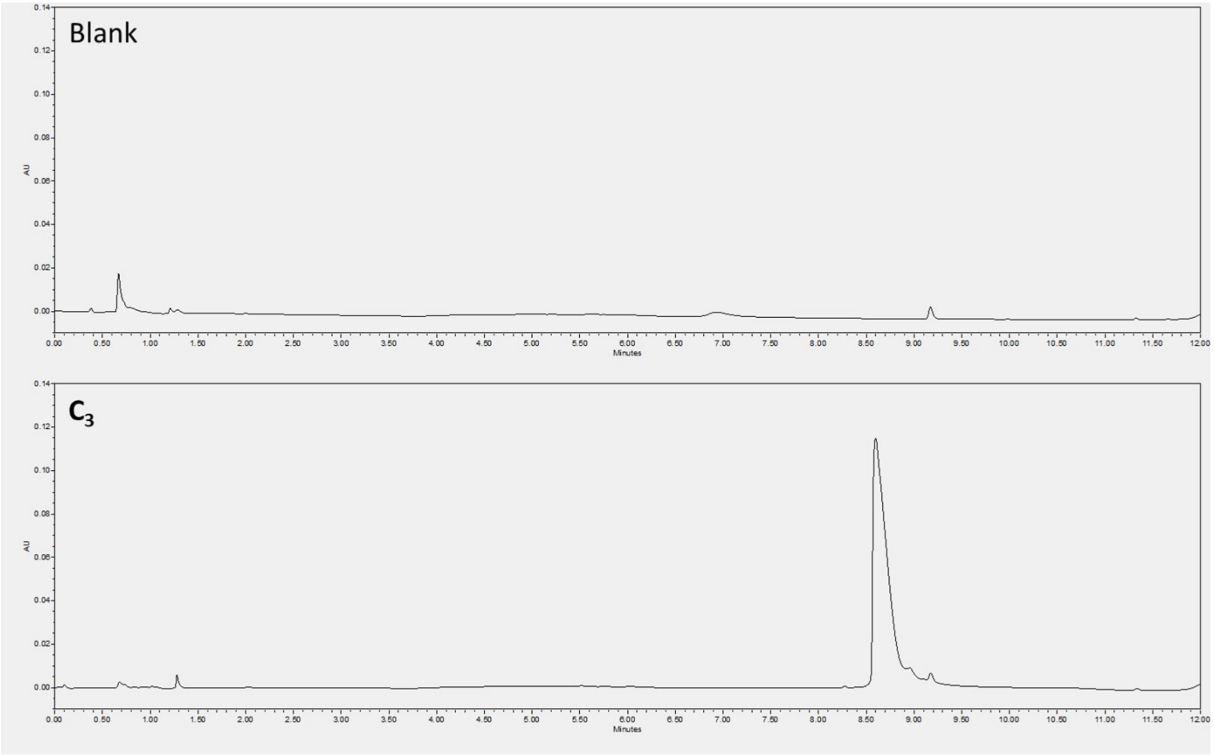

Fig. S7 Analytical RP-HPLC chromatogram of conjugate $\mathbf{C_3}$. Free TLR7/8L derivative, which elutes at an earlier retention time than the conjugate peak, was not observed.

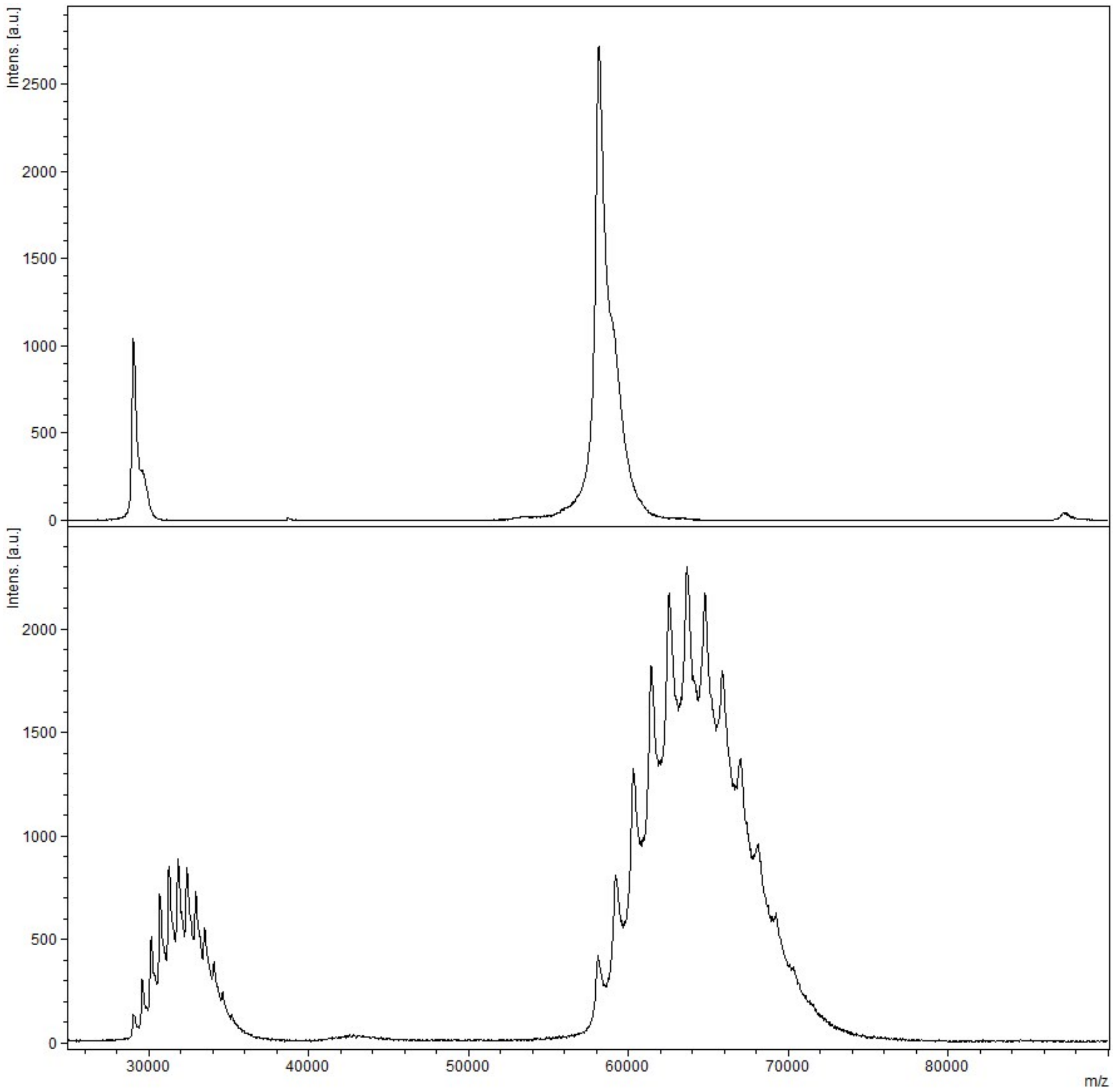

Fig. S8 MALDI-TOF mass spectrum of unmodified CRM-197 and conjugate $\mathbf{C_3}$ (top to bottom). Rightmost peaks correspond to $[M + H]^+$ ions; leftmost peaks correspond to $[M + 2H]^{2+}$ ions.

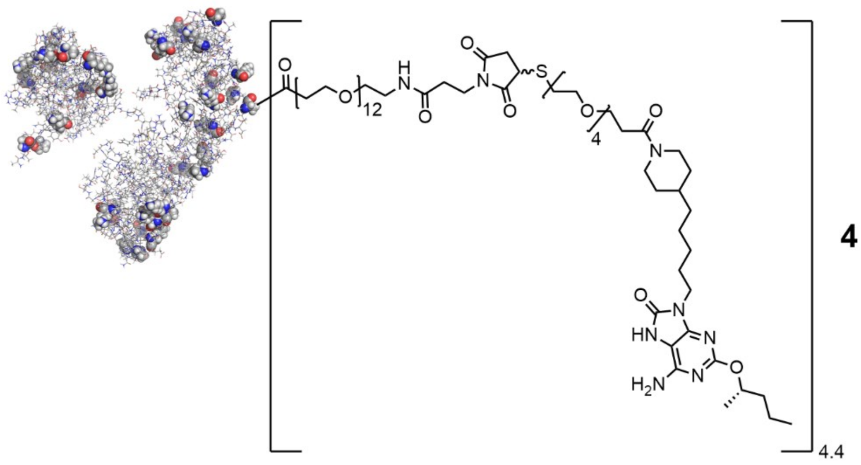

C_4: CRM-197-4

Conjugate $\mathbf{C_4}$ was synthesized in two parts: a) compound **4** was synthesized from **4b** *in situ* and b) the stock solution of compound **4** was added to CRM-197 to create conjugate $\mathbf{C_4}$.

Part a: compound **4** was created *in situ* and used immediately after preparation. In a plastic vial, 3.4 mg (0.0039 mmol, 1.0 equiv) of SM(PEG)$_{12}$ (succinimidyl-[(*N*-maleimidopropionamido)-dodecaethyleneglycol] ester, ThermoFisher) were dissolved in 168.9 μL of anhydrous DMF. In a separate plastic vial, a 10.0 mg/mL solution of **4b** was prepared by dissolving 6.0 mg of **4b** in 600 μL of anhydrous DMF. The SM(PEG)$_{12}$ solution was titrated with **4b** and monitored by LC/MS. This was accomplished by adding a total of 300 μL (3.0 mg, 0.0039 mmol) of the **4b** stock solution to the SM(PEG)$_{12}$ stock solution in the presence of 2.0 μL (3.0 equiv) of DIPEA. After each addition of compound **4b**, the reaction was allowed to react at RT for 15 min without stirring after some initial vortexing. This titration resulted in the full *in situ* conversion of the SM(PEG)$_{12}$ starting material to **4** (quantitative). Although used immediately after preparation, the stock solution of **4** exhibited good stability for weeks at –20 °C. HRMS ($C_{69}H_{118}N_9O_{26}S^+$ = $[M + H]^+$): calculated = 1520.7903; found = 1520.7899.

Part b: in a plastic conical vial, a 1.0 mg/mL solution of 1.8 mg of CRM-197 (0.000031 mmol) in 10 mM PB pH 7.2 was prepared from a more concentrated stock solution of CRM-197. While vortexing gently, 327 μL (0.0026 mmol) of the stock solution of **4** were slowly added to the protein vial. The reaction appeared clear and colorless and was allowed to proceed at RT without stirring for 45 min. Conveniently, the reaction progress could be monitored by MALDI-TOF MS. Four 10 mL Zeba Spin Desalting Columns (7 KDa cutoff) equilibrated with 10 mM PB pH 7.2 were used to purify the bioconjugate. Due to the relatively large molecular weight of the TLR7/8L derivative, the elimination of free, unconjugated compound **4** was confirmed using analytical RP-HPLC (using the equipment, mobile phases, and gradient described in Section 1). MALDI-TOF MS was used to determine an adjuvant copy # of 4.4. The clear and colorless formulation was sterile filtered. A BCA assay kit was utilized to determine the total mass and yield of conjugate $\mathbf{C_4}$ (1.5 mg, 73%).

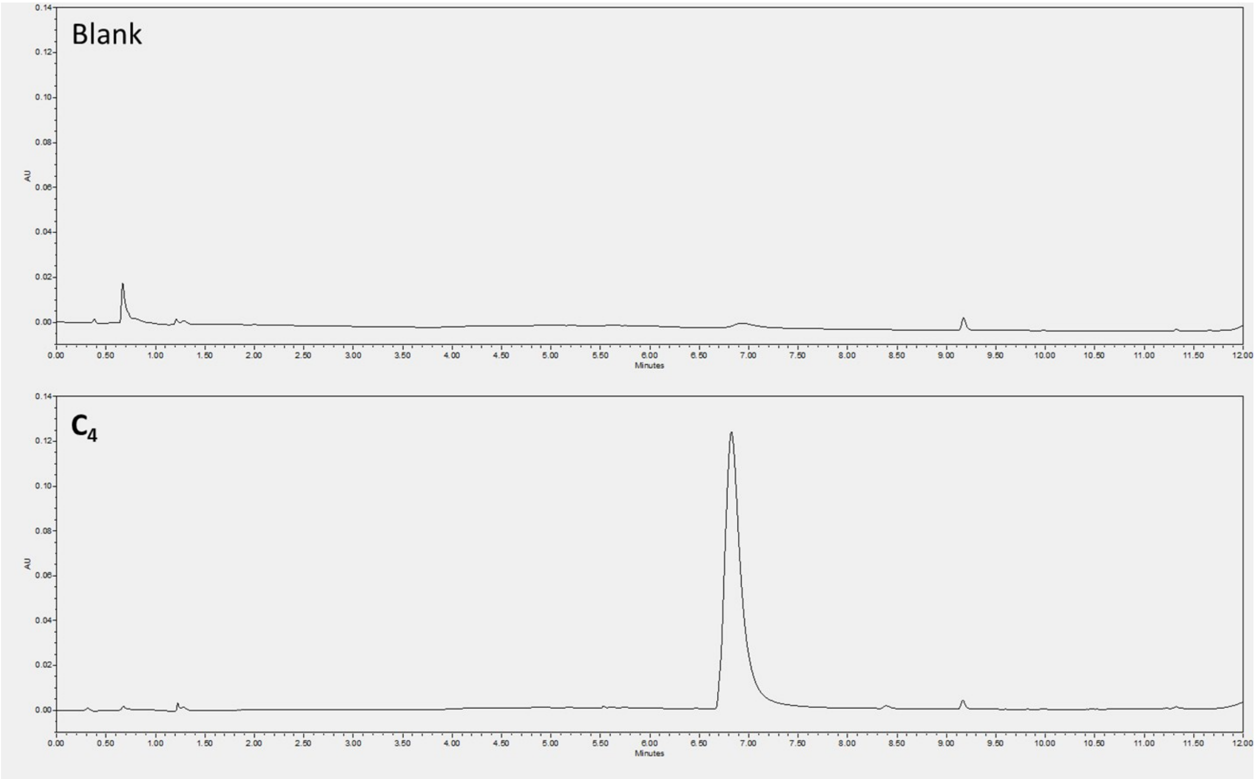

Fig. S9 Analytical RP-HPLC chromatogram of conjugate $\mathbf{C_4}$. Free TLR7/8L derivative, which elutes at an earlier retention time than the conjugate peak, was not observed.

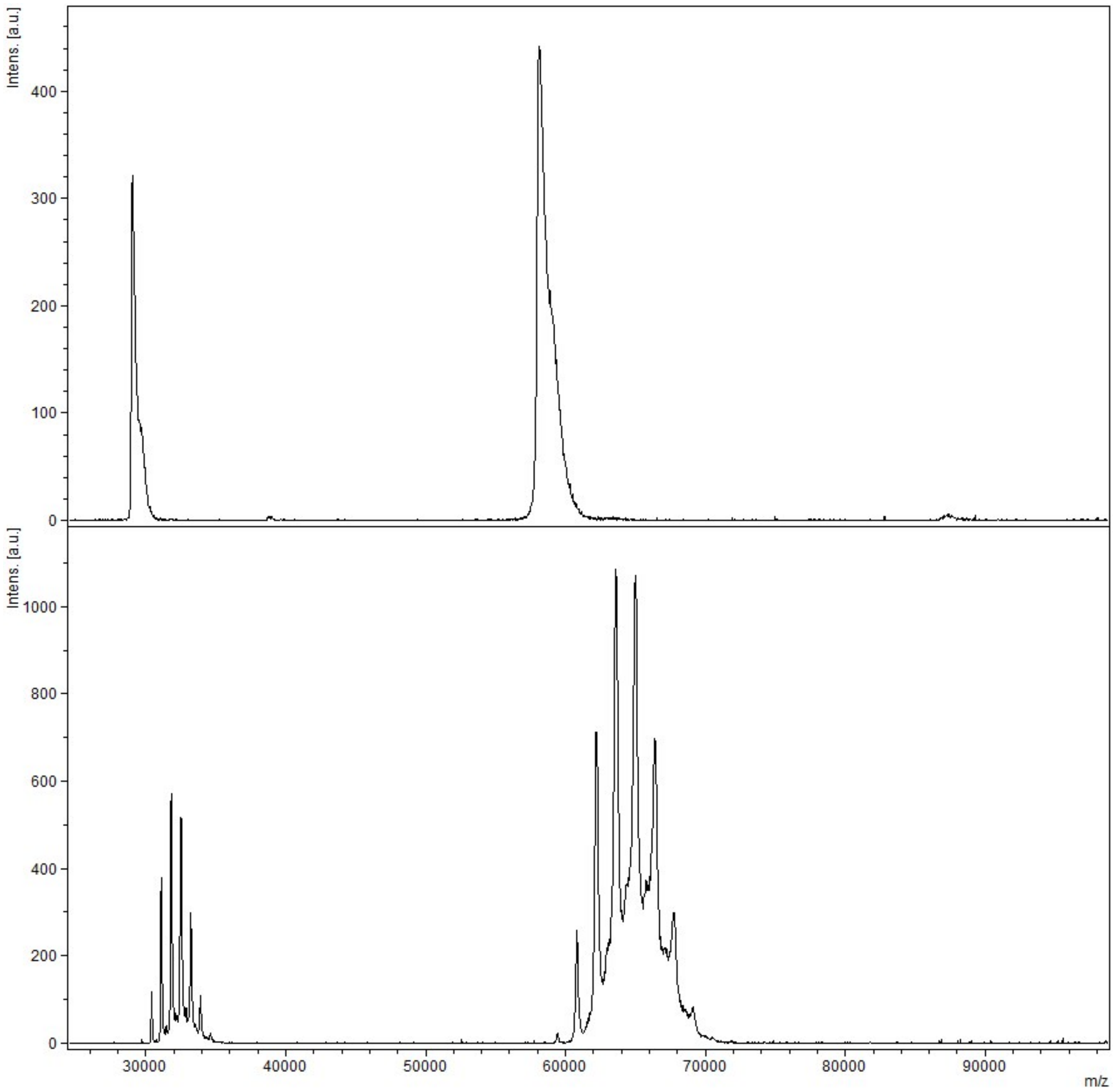

Fig. S10 MALDI-TOF mass spectrum of unmodified CRM-197 and conjugate $\mathbf{C_4}$ (top to bottom). Rightmost peaks correspond to $[M + H]^+$ ions; leftmost peaks correspond to $[M + 2H]^{2+}$ ions.

Section 3: TLR7/8L water solubility comparison

Compound	Intrinsic solubility (mg/mL)	Solubility at pH 7.4 (mg/mL)
R848	1.1	1.1
UM-3007[a]	0.2	2.2
UM-3013[a]	0.2	0.2
UM-3006[a]	0.0	0.0

Table S2 Calculated water solubilities of TLR7/8Ls upon amide functionalization. R848 was used as a comparator. Predicted solubilities were calculated using MarvinSketch 19.27.0 and confirmed by visual observations.

[a]Amine handle was substituted with a formamidyl group to approximate the effect of amide functionalization.

Section 4: *In vitro* immunological methods and supplementary data

HEK-293 TLR7 and TLR8 reporter assays: human TLR7- or TLR8-expressing HEK-293 reporter cells were obtained from Novus Biologicals (TLR7) or Invivogen (TLR8, San Diego, CA). These cells constitutively express either TLR7 or TLR8 as well as NFκB-driven production of secreted embryonic alkaline phosphatase (SEAP) as a readout for TLR7 or TLR8 signaling. Cells were cultured according to the manufacturer's instructions in DMEM with 10% FBS, 1% Penicillin-Streptomycin-Glutamine solution (Hyclone Cat #: SV30082.01), 0.1% 2-mercaptoethanol (Gibco Cat #: 21985.023), and selection antibiotics. Cells were plated at a density of 1×10^5 cells/well in flat bottom 96-well plates, and duplicate wells were incubated for 42–48 h (hTLR7) or 18–24 h (hTLR8) at 37 °C with the indicated concentrations of various TLR7/8Ls. Cell supernatants were harvested and analyzed for SEAP via the manufacturer's instructions using a QuantiBlue kit (Invivogen) by reading of the optical density (OD) at 650 nm with a microplate reader. Data are expressed as the average fold change in OD over that of wells containing vehicle-treated cells.

Human peripheral blood mononuclear cell (hPBMC) isolation and stimulation: human blood was obtained from anonymous healthy adult donors through a protocol approved by a University of Montana Institutional Review Board (IRB). hPBMCs were separated from whole blood via density gradient separation using Histopaque 1,077 (Sigma). For hPBMC-based assays, 5×10^5 cells were plated in 96-well flat bottom TC plates in complete medium (RPMI 1640 supplemented with 10% FBS, 292 μg/ml glutamine, 100 U/ml penicillin, 100 μg/ml streptomycin, and 55 μM β-mercaptoethanol). Cells were treated with the indicated compound concentrations

for 24 h. Supernatants were harvested and stored at −20 °C until assayed. Cytokines were detected by ELISA for TNFα (DuoSet, R&D Systems) or IFNα (PBL Assay Science).

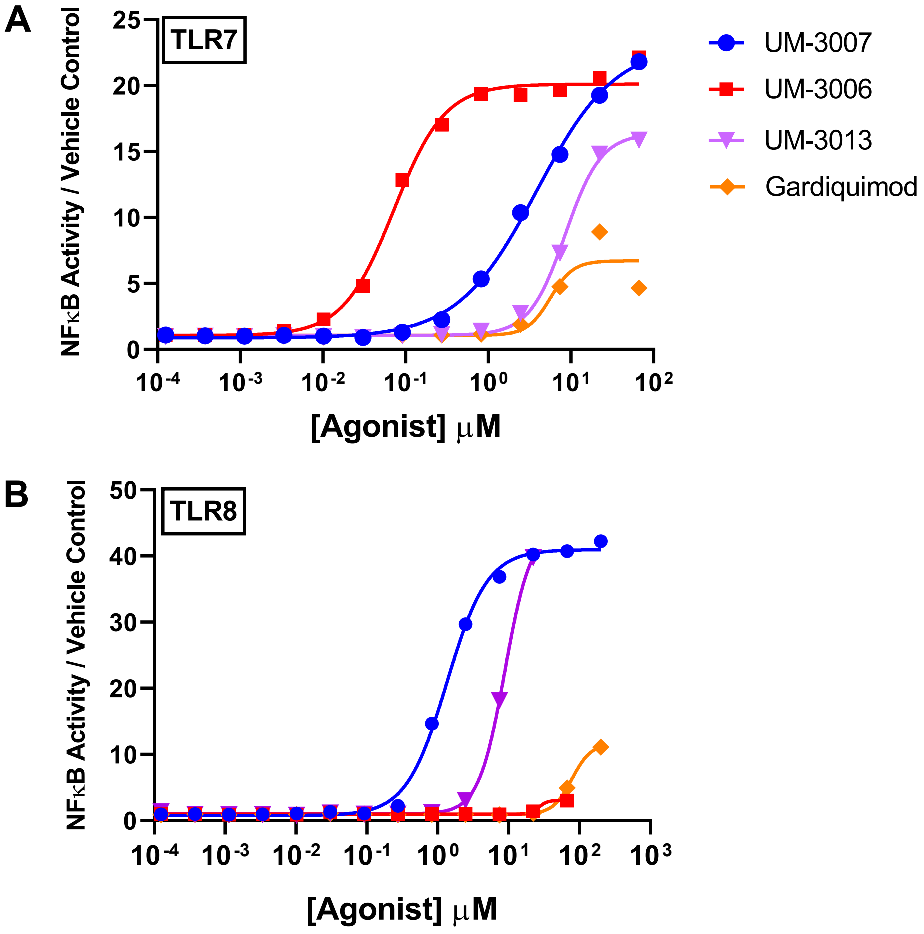

Fig. S11 Four TLR7/8Ls used as adjuvants in these studies were tested for signaling through TLR7 (A) and TLR8 (B) using HEK-293 reporter cells expressing either human TLR7 or TLR8 and NF-κB-driven production of secreted embryonic alkaline phosphatase (SEAP). Cells were exposed to a range of concentrations of the agonists for either 48 (A) or 24 (B) hours, followed by measurement of SEAP in the culture supernatants. Data are expressed as the average of the fold

change in SEAP production for duplicate wells over that of the vehicle-only treated cells. Lines represent the four parameter least squares fit as determined by GraphPad Prism 8. Commercially available Gardiquimod was used a positive control.

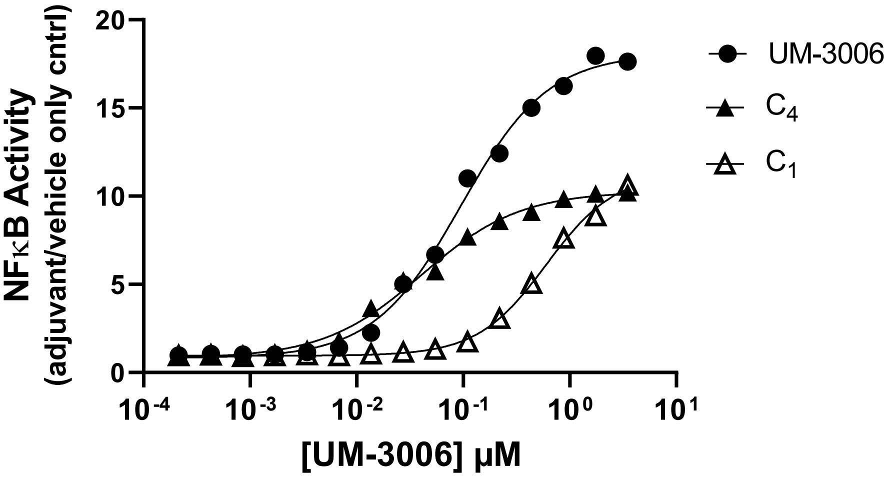

Fig. S12 The activities of conjugates $\mathbf{C_4}$ and $\mathbf{C_1}$ were compared to unconjugated UM-3006 on HEK-293 TLR7 reporter cells. The detection of SEAP in cell supernatants after a 48 h exposure to the indicated concentrations of UM-3006 (either free or conjugated) was measured as an indication of TLR7 signaling. Data points are the average of duplicate wells; lines represent the four parameter least squares fit as determined by GraphPad Prism 8.

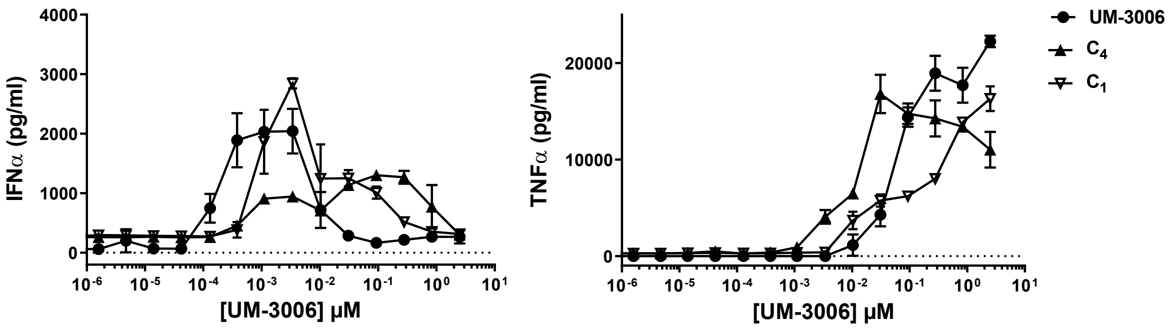

Fig. S13 Cytokine production by hPBMCs upon exposure to the indicated concentration of either free UM-3006 or a CRM-197-UM-3006 conjugate (**C_4** or **C_1**). Cells were incubated for 24 h, then cell supernatants were collected and assayed by ELISA for IFNα and TNFα. Data shown are representative of three independent experiments with cells from different donors. Data points are the average of duplicate wells.

Section 5: *In vivo* immunological methods and supplementary data

Vaccinations: female 6–8 week old BALB/c mice (Envigo) were housed in an AAALAC-accredited facility, and all procedures were performed in accordance with a UM IACUC-approved animal use protocol. Mice were immunized three times, 14 days apart, by intramuscular injection in the left hind limb. Fourteen days after the primary and secondary injections, submandibular bleeds were performed, and serum was separated using Microtainer Serum Separator Tubes (BD Biosciences) then stored at 4 ˚C for the short term or –20 ˚C for longer storage until use. Five days after the tertiary injection, the mice were euthanized, and the spleens and draining (inguinal and popliteal) lymph nodes (DLNs) from the side of vaccination were harvested into cold PBS.

ELISA for antigen-specific antibody quantification: serum was analyzed via ELISA for antigen-specific (either BSA or CRM-197) total IgG, and depending on the experiment, IgG1 and IgG2a antibody titers. Serum was diluted according to the expected antibody response (between 1:10 and 1:500). MaxiSorp ELISA plates (Nunc) were coated with 100 μl of 12.5 μg/mL CRM-197 or 20 μg/mL BSA overnight at RT. Plates were washed 3 × with 1% Tween-20 in PBS (PBS-T; Sera Care cat #: 54600-0026) then blocked with SuperBlock (ScyTek Laboratories cat #: AAA999) at 37 ˚C for 1 h. Following removal of SuperBlock, plates were incubated with serum serial diluted in SuperBlock (eight dilutions per sample) for 1 h for a CRM ELISA and 2 h for a BSA ELISA, washed three times with PBS-T, then incubated with anti-mouse IgG-, and depending on the experiment, IgG1- or IgG2a-HRP secondary antibodies in SuperBlock for 1 h for a CRM ELISA and 2 h for a BSA ELISA (1:500 and 1:2000, respectively; All Southern Biotech). Secondary antibodies were detected by addition of TMB Substrate (BD Biosciences) for 1 h, followed by measurement of OD

at 650 nm using a Molecular Devices SpectraMax 190 microplate reader. Serum antibody titers are reported as the dilution factor of each sample that yielded an OD of 0.300 calculated by determining the best fit line to the absorbance values of the serial dilutions and then extrapolating (XLFit, IDBS).

Ex vivo lymphocyte restimulation and cell-mediated immunity analysis: the DLNs on the side of immunization from each mouse were disrupted by gentle agitation between the frosted ends of sterilized microscope slides (VWR cat #: 16004-372) in cold HBSS. Cells were then pelleted by centrifugation at 400 × g at 4 ˚C for 5 min and resuspended in 1 mL of cold HBSS for counting. Spleens were disrupted by gentle agitation against a sterile 100 μm filter in cold PBS. Cells were pelleted at 400 × g for 10 minutes at 4 ˚C, followed by red blood cell lysis by incubation with red blood cell lysis buffer (BioLegend cat #: 420301) for 5 min at RT, followed by washing with cold HBSS. Cells were resuspended in 10 mL of cold HBSS filtered through a sterile 100 μm filter and counted by a hemocytometer. Cells were then pelleted and resuspended in an appropriate volume of cRPMI for plating. Lymph-node cells were plated at 2×10^6 cells/well and splenocytes were plated at 5×10^6 cells/well of a flat-bottom 96-well plate in the presence of 10 μg/mL whole antigen (CRM-197 or BSA) in a total volume of 200 μL. Plates were incubated at 37 °C with 5% CO_2 for 72 h. Cell supernatants were then collected and stored at –20 °C until assayed.

Multiplex ELISA analysis of cell supernatants: supernatants collected from *ex vivo* DLN cells restimulated with antigen for 72 h were tested for IFNγ, TNFα, IL-2, IL-17A, IL-10, and IL-5 using the U-Plex multiplex ELISA kit (Meso Scale Diagnostics, Rockville, MD) following the manufacturer's instructions.

Statistical analyses: statistics were analyzed using GraphPad Prism 8. In Figs. 3, S15, and S16, the Kruskal-Wallis test for non-parametric data was used to determine significance. In Fig. S14, one-way ANOVA was used to determine variances in means between formulation groups with Tukey's test for post-hoc comparison of means (p < 0.05 *, < 0.01 **, <0.001 *** < 0.0001 ****).

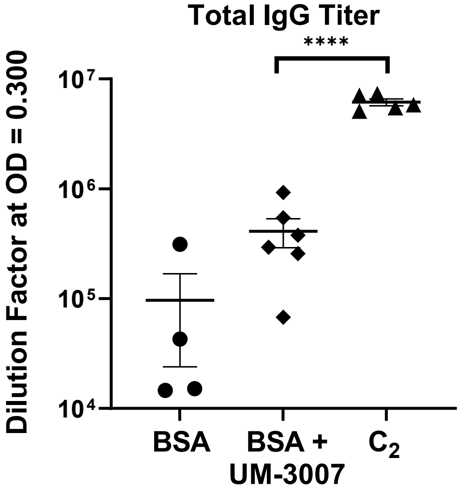

Fig. S14 Total IgG anti-BSA serum antibody titers 14 days after secondary immunization with conjugate $\mathbf{C_2}$ vs. equimolar amounts of free BSA antigen and UM-3007. Each symbol represents an individual mouse.

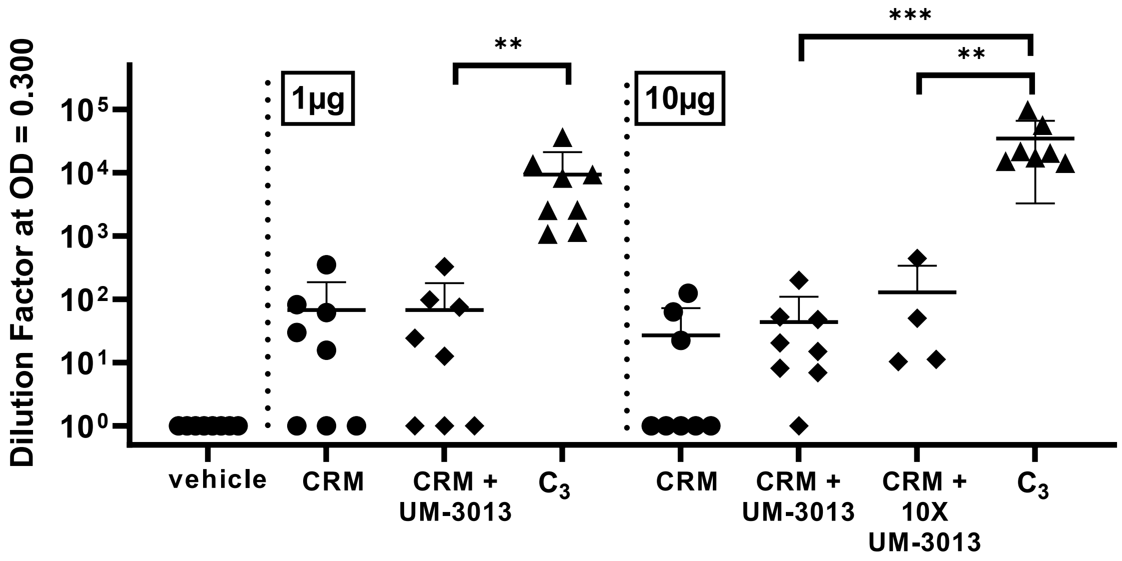

Fig. S15 Total IgG anti-CRM-197 serum antibody responses 14 days post-secondary vaccination with conjugate **C_3** as compared to an equimolar mixture of CRM-197 and UM-3013. With mice immunized with 10 μg of CRM-197, conjugation significantly increased the antibody titer even when ten times the amount of adjuvant was added. Each symbol represents an individual mouse.

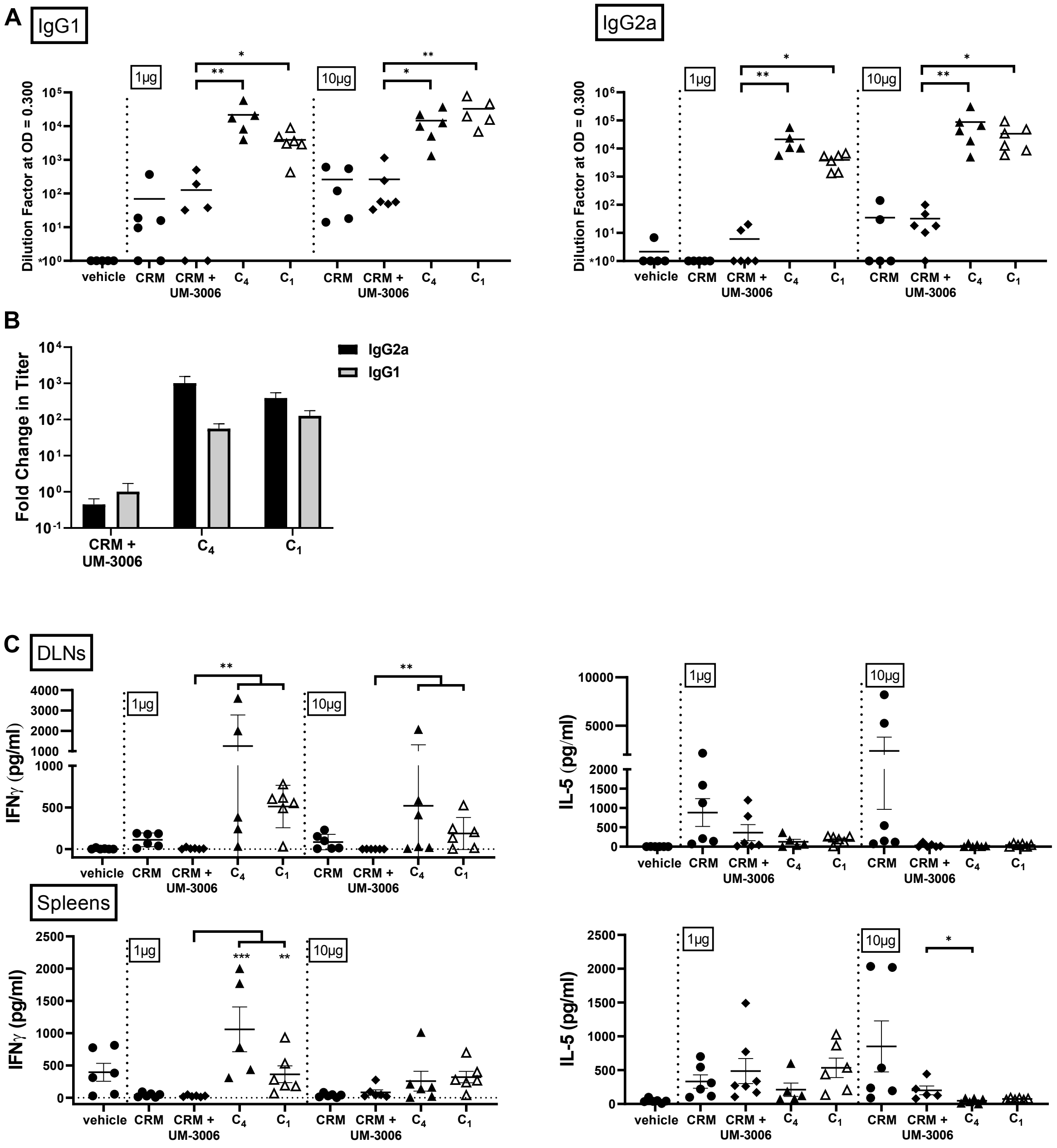

Fig. S16 Immune responses in mice vaccinated with conjugate **C_4** or **C_1** compared to an equimolar mixture of free antigen and adjuvant. A) IgG1 and IgG2a anti-CRM-197 serum antibody responses 14 days following secondary vaccination. B) Antibody titers from the 10 μg groups shown in A were normalized by expressing them as the fold-change over the mean of the corresponding antigen alone (CRM-197) group. This allows the comparison of the boost in antibody titer for

each isotype. Data shown are the mean +/- SEM for each group of mice. C) IFNγ and IL-5 concentrations in supernatants of *ex vivo* DLN (top) and spleen (bottom) cells stimulated for 72 h with whole antigen. Each symbol represents an individual mouse. **$p < 0.005$

References

1. H. G. Bazin, Y. Li, J. K. Khalaf, S. Mwakwari, M. T. Livesay, J. T. Evans and D. A. Johnson, *Bioorganic & medicinal chemistry letters*, 2015, **25**, 1318-1323.
2. J. T. Evans, L. S. Bess, S. C. Mwakwari, M. T. Livesay, Y. Li, V. Cybulski, D. A. Johnson and H. G. Bazin, *ACS omega*, 2019, **4**, 15665-15677.
3. H. G. Bazin, L. S. Bess, M. T. Livesay, Y. Li, V. Cybulski, S. M. Miller, D. A. Johnson and J. T. Evans, *Bioorganic & Medicinal Chemistry Letters*, 2020, **30**, 126984.
4. Lindstrom, K. J.; Martin, H.; Merryl, B. A.; Rice, M. J.; Wurst, J. R.; Haraldson, C. A.; Kshirsagar, T.; Heppner, P. D.; Niwas, S.; Griesgraber, G. W.; Radmer, M. R. Aryloxy and arylalkyleneoxy substituted imidazoquinolines. WO/2005/020999.
5. U. Wille-Reece, B. J. Flynn, K. Loré, R. A. Koup, R. M. Kedl, J. J. Mattapallil, W. R. Weiss, M. Roederer and R. A. Seder, *Proceedings of the National Academy of Sciences*, 2005, **102**, 15190-15194.
6. U. Wille-Reece, C. Y. Wu, B. J. Flynn, R. M. Kedl and R. A. Seder, *J Immunol*, 2005, **174**, 7676-7683.
7. K. Kastenmuller, U. Wille-Reece, R. W. Lindsay, L. R. Trager, P. A. Darrah, B. J. Flynn, M. R. Becker, M. C. Udey, B. E. Clausen, B. Z. Igyarto, D. H. Kaplan, W. Kastenmuller, R. N. Germain and R. A. Seder, *J Clin Invest*, 2011, **121**, 1782-1796.
8. N. M. Shukla, T. C. Lewis, T. P. Day, C. A. Mutz, R. Ukani, C. D. Hamilton, R. Balakrishna and S. A. David, *Bioorg Med Chem Lett*, 2011, **21**, 3232-3236.
9. L. Fili, A. Vultaggio, E. Cardilicchia, C. Manuelli, A. Casini, F. Nencini, L. Maggi, S. Pratesi, G. Petroni, F. Boscaro, A. Guarna, E. G. Occhiato, S. Romagnani, E. Maggi and P. Parronchi, *J Allergy Clin Immunol*, 2013, **132**, 84-92.
10. Y. Feng, M. N. Forsell, B. Flynn, W. Adams, K. Lore, R. Seder, R. T. Wyatt and G. B. Karlsson Hedestam, *Virology*, 2013, **446**, 56-65.
11. S. Vecchi, S. Bufali, T. Uno, T. Wu, L. Arcidiacono, S. Filippini, F. Rigat and D. O'Hagan, *Eur J Pharm Biopharm*, 2014, **87**, 310-317.
12. D. Gao, Y. Liu, Y. Diao, N. Gao, Z. Wang, W. Jiang and G. Jin, *ACS Med Chem Lett*, 2015, **6**, 249-253.
13. D. Gao, J. Zeng, X. Wang, Y. Liu, W. Li, Y. Hu, N. Gao, Y. Diao, Z. Wang, W. Jiang, J. Chen and G. Jin, *Eur J Med Chem*, 2016, **120**, 111-120.
14. D. S. Wilson, S. Hirosue, M. M. Raczy, L. Bonilla-Ramirez, L. Jeanbart, R. Wang, M. Kwissa, J. F. Franetich, M. A. S. Broggi, G. Diaceri, X. Quaglia-Thermes, D. Mazier, M. A. Swartz and J. A. Hubbell, *Nat Mater*, 2019, **18**, 175-185.
